# Odd Willis coupling induced by broken time-reversal symmetry

**DOI:** 10.1038/s41467-021-22745-5

**Published:** 2021-05-10

**Authors:** Li Quan, Simon Yves, Yugui Peng, Hussein Esfahlani, Andrea Alù

**Affiliations:** 1 Department of Electrical and Computer Engineering, The University of Texas at Austin, Austin, TX USA; 2 Photonics Initiative, Advanced Science Research Center, City University of New York, New York, NY USA; 3 Physics Program, Graduate Center, City University of New York, New York, NY USA

**Keywords:** Mechanical engineering, Acoustics

## Abstract

When sound interacts with geometrically asymmetric structures, it experiences coupling between pressure and particle velocity, known as Willis coupling. While in most instances this phenomenon is perturbative in nature, tailored asymmetries combined with resonances can largely enhance it, enabling exotic acoustic phenomena. In these systems, Willis coupling obeys reciprocity, imposing an even symmetry of the Willis coefficients with respect to time reversal and the impinging wave vector, which translates into stringent constraints on the overall scattering response. In this work, we introduce and experimentally observe a dual form of acoustic Willis coupling, arising in geometrically symmetric structures when time-reversal symmetry is broken, for which the pressure-velocity coupling is purely odd-symmetric. We derive the conditions to maximize this effect, we experimentally verify it in a symmetric subwavelength scatterer biased by angular momentum, and we demonstrate the opportunities for sound scattering enabled by odd Willis coupling. Our study opens directions for acoustic metamaterials, with direct implications for sound control, non-reciprocal scattering, wavefront shaping and signal routing, of broad interest also for nano-optics, photonics, elasto-dynamics, and mechanics.

## Abstract (web summary)

Exploiting Willis coupling in acoustic metamaterials with designed geometrical asymmetries opens up new opportunities related to sound control and manipulation. Here, the authors report a dual form of Willis coupling in geometrically symmetric acoustic scatterers.

## Introduction

Symmetries and symmetry breaking play a key role in a wide range of natural phenomena^1,2^. According to Noether’s theorem, any symmetry in a physical system corresponds to a conservation law, and these laws fundamentally define material responses and constitutive relations. For example, in elastodynamics energy conservation restricts the elastic modulus tensor to be even symmetric, ${C}_{ijmn}={C}_{mnij}$^3^, while in fluid mechanics Onsager relations require the viscosity to be even symmetric ${\eta }_{ijkl}={\eta }_{klij}$^4^. Viscosity can support an odd term when time reversal symmetry is broken, losing its dissipative nature^5–8^. Similarly, odd elasticity has been recently discussed in active solids where energy is not conserved, enabling among other features exotic elastic wave propagation in overdamped media^9^. These forms of material constitutive relations based on broken symmetries have been opening interesting directions for exotic wave matter interactions, and the quest to realize these phenomena in engineered materials and metamaterials is ongoing.

In acoustics, geometrical asymmetries in the constituent elements of metamaterials have been at the basis of the recent interest in Willis coupling^10–16^, the analog of bianisotropy in optics and electromagnetics^17^, which describes the coupling between pressure and particle velocity. Although the emergence of Willis coupling in asymmetric structures has been first discussed almost 40 years ago, its effects have been broadly considered a higher-order perturbation of the direct response of materials separately to pressure and velocity^18–20^. Recently, our group showed that the magnitude of Willis coupling can become very strong in suitably designed metamaterials in which tailored geometrical asymmetries are combined with resonances^21,22^. In electromagnetics, bianisotropy has been exploited to produce largely asymmetric absorption^23,24^, topological phenomena^25^, and to overcome the limitations of gradient metasurfaces for wavefront engineering^26^, among several other opportunities. Similarly, Willis metamaterials hold the promise to open analogous opportunities for elastic and sound waves^21,27–30^. Onsager relations pose restrictions on the nature of these Willis coefficients, requiring that they are even symmetric with respect to time-reversal and to the impinging wave vector. In the following, we introduce and experimentally observe a dual form of Willis acoustic coupling, which is inherently odd-symmetric in nature with respect to time reversal, and it arises from non-reciprocal sound interactions in suitably tailored devices.

## Results

### Even and odd Willis coupling

For a subwavelength Willis scatterer, the reaction to an impinging sound wave consists in acoustic radiation dominated by the monopole *M* and the dipole ${\bf{D}}$ scattering contributions. These quantities are proportional to the local pressure *p* and velocity ${\bf{v}}$ through the polarizability tensor ${\mathop{\boldsymbol{\alpha }}\limits^{\leftrightarrow}}$:^21^

1 $$\left(\begin{array}{c}M\\ {\bf{D}}\end{array}\right)=\left(\begin{array}{cc}{\alpha }^{pp} & {\mathop{\boldsymbol{\alpha }}\limits^{\leftrightarrow}}{\,\!}^{pv}\\ {\mathop{\boldsymbol{\alpha }}\limits^{\leftrightarrow}}{\,\!}^{vp} & {\mathop{\boldsymbol{\alpha }}\limits^{\leftrightarrow}}{\,\!}^{vv}\end{array}\right)\left(\begin{array}{c}p\\ {\bf{v}}\end{array}\right),$$

where ${\alpha }^{pp}$ and ${\mathop{\boldsymbol{\alpha }}\limits^{\leftrightarrow}}{\,\!}^{vv}$ represent the direct polarizability coefficients, relating pressure to monopole and velocity to dipole, as in conventional acoustic scattering, while ${\mathop{\boldsymbol{\alpha }}\limits^{\leftrightarrow}}{\,\!}^{vp}$ and ${\mathop{\boldsymbol{\alpha }}\limits^{\leftrightarrow}}{\,\!}^{pv}$ are the cross-coupling polarizabilities responsible for Willis coupling. In (1), we have normalized the units as defined in Supplementary Information 1, so that all elements of the polarizability tensor share the same dimensionality. The off-diagonal elements, known as Willis coefficients, arise in structures that break spatial inversion symmetry with respect to the propagation direction^15^, and are enhanced when suitably tailored geometrical asymmetries are combined with strong resonances^21^. They satisfy the symmetry condition ${\mathop{\boldsymbol{\alpha }}\limits^{\leftrightarrow}}{\,\!}^{vp}=-{\mathop{\boldsymbol{\alpha }}\limits^{\leftrightarrow}}{\,\!}^{pvT}$, which is required to ensure an even-symmetric response with respect to the impinging wave vector and time-reversal.

**Fig. 1:**
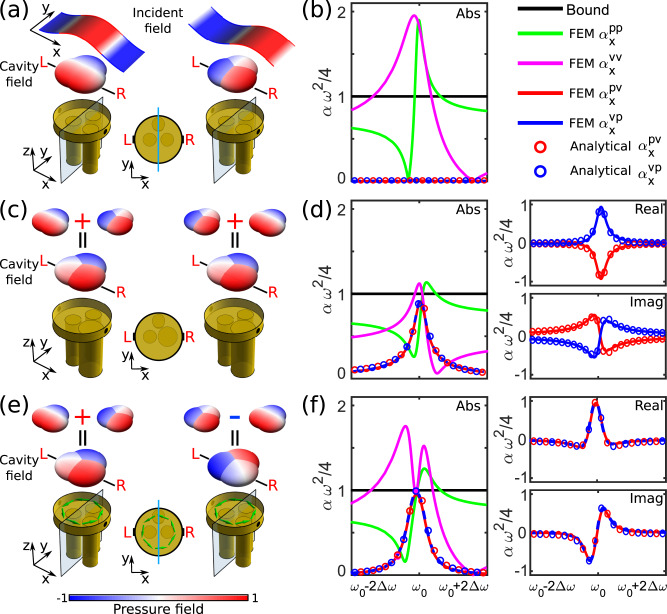
Even and odd Willis inclusions. **a** Induced pressure field distribution inside the scatterer for a local pressure (left) and velocity (right) standing wave excitation, in the case of a mirror-symmetric scatterer. The colored blobs here and in the other panels indicate the induced pressure inside each of the cavities loading the scatterer. **b** Corresponding polarizability dispersion around the resonance frequency. **c** Similar to A, but for an even Willis scatterer, obeying time-reversal symmetry. The induced field distributions correspond to a superposition of monopolar and dipolar fields, yielding identical field distributions for pressure and velocity excitations. **d** Corresponding polarizability dispersion around the resonance frequency. **e** Similar to A, but for an odd Willis scatterer, obeying mirror symmetry along the propagation direction, but biased by a rotating flow. In this case, the pressure-induced fields are identical to the even Willis scenario, but when excited by a velocity field the zero pressure fields shift from the left to the right cavity. **f** Corresponding polarizability dispersion around the resonance frequency.

In this study, we introduce and experimentally observe a dual form of Willis coupling, inherently odd-symmetric in nature, arising when time-reversal symmetry is broken in a subwavelength scatterer, but mirror symmetry with respect to the incoming wave propagation is preserved, for which ${\mathop{\boldsymbol{\alpha }}\limits^{\leftrightarrow}}{\,\!}^{vp}={\mathop{\boldsymbol{\alpha }}\limits^{\leftrightarrow}}{\,\!}^{pvT}$. As schematically shown in Fig. 1a, we consider a circular scatterer loaded by three small cylindrical cavities located in a mirror-symmetric configuration with respect to the *x* axis, with two oppositely facing outlets (L and R) in the *x*-direction that allow sound to interact with the cavities. Because of the outlet location, we expect a strong acoustic response in the subwavelength limit only for waves impinging along *x*, yielding a dipolar response polarized in the same direction, ${D}_{x}$. Under these assumptions, Eq. (1) is simplified to

2 $$\left(\begin{array}{c}M\\ {D}_{x}\end{array}\right)=\left(\begin{array}{cc}{\alpha }^{pp} & {\alpha }_{x}^{pv}\\ {\alpha }_{x}^{vp} & {\alpha }_{xx}^{vv}\end{array}\right)\left(\begin{array}{c}p\\ {v}_{x}\end{array}\right).$$

Given its mirror symmetry, when the subwavelength scatterer is excited symmetrically from the two sides with a field pressure maximum at its location, as shown in Fig. 1a (left), the induced pressure distribution in the three cavities, shown in the same panel as colored blobs, necessarily obeys mirror-symmetry, and hence the scattered fields at the outlets L and R are symmetric, i.e., purely monopolar. Conversely, if the relative phase of the impinging waves is shifted by $\pi /2$, such that the pressure field has a node at the scatterer location, as in Fig. 1a (right), the scatterer is dominantly excited by the local velocity, the induced modal distribution becomes anti-symmetric with *x*, and the scattered field is purely dipolar. It follows that the Willis coupling coefficients for this scenario ${\alpha }_{x}^{pv}={\alpha }_{x}^{vp}=0$, as confirmed in the retrieved polarizability elements shown in Fig. 1b, calculated with finite-element simulations as detailed in the Methods Section. The direct terms, as expected, peak around the cavity resonance frequency.

Willis coupling can be introduced by breaking the mirror symmetry with respect to the direction of sound propagation, for instance by making the right cavity larger than the left one, as in Fig. 1c. In this case, the response at outlets L and R is generally no longer symmetric for a pressure excitation (left), nor anti-symmetric for a velocity excitation (right). Both excitations therefore support the superposition of monopolar and dipolar distributions inside the scatterer, implying a finite Willis coupling. Indeed, Fig. 1d shows the retrieved polarizability coefficients, which, in line with the findings in^21^, show a resonant dispersion of the Willis coefficients. Because of the optimal design of the scatterer of Fig. 1c, they actually hit their maximum possible value $|{\alpha }_{x}^{pv}|=|{\alpha }_{x}^{vp}|=4{\omega }^{-2}$ at the scatterer resonance. This bound, indicated by the black solid lines in Fig. 1, generally stems from energy conservation, and it therefore applies to any passive scatterer^21^ (Supplementary Information 2). The field plots in Fig. 1c correspond to the excitation at this resonance frequency, for which the Willis coefficients are maximum. Under this condition, the fields induced by pressure and velocity excitations are identical, stemming from their even-symmetric nature with respect to time-reversal, ${\alpha }_{x}^{vp}=-{\alpha }_{x}^{pv}$, and from their magnitude being equal to the direct polarizability terms at resonance when $|{\alpha }_{x}^{pv}|=|{\alpha }_{x}^{vp}|=4{\omega }^{-2}$. These features are confirmed in the retrieved polarizability coefficients in Fig. 1d, where we also show the real and imaginary parts of the Willis coefficients, confirming their even symmetry with respect to time reversal.

We now introduce a dual mechanism to induce Willis coupling, as shown in Fig. 1e. Instead of breaking mirror symmetry, as in panel c, we introduce a circulating air flow in the symmetric scatterer of panel a. The system has zero even Willis coupling because of its mirror symmetry along *x*, but the air flow (green arrows) biases the system with an odd quantity under time-reversal^31,32^, which, when combined with a suitable scatterer design, can again maximally couple the response to pressure and velocity excitations. Importantly, in this dual scenario the superposition of the two modal distributions switches sign, as shown in the figure, yielding a drastically different modal distribution inside the scatterer, which translates into scattering with an odd-symmetric Willis response, ${\alpha }_{x}^{vp}={\alpha }_{x}^{pv}$, as confirmed in the retrieved polarizability tensor in Fig. 1f. The odd Willis coefficients still obey the bound $|{\alpha }_{x}^{pv}|\le 4{\omega }^{-2}$, $|{\alpha }_{x}^{vp}|\le 4{\omega }^{-2}$ (Supplementary Information 2) and hit this bound at the scatterer resonance for our optimal geometry, with diagonal and off-diagonal polarizability elements equal in magnitude to $4{\omega }^{-2}$ (Supplementary Information 2, 3), because the system remains conservative as long as the air flow is much slower than the velocity of sound. At this resonance, the pressure-induced fields are identical to the even Willis scenario, but when excited by a velocity field the zero pressure field shifts from the left to the right cavity.

In order to get a deeper insight into this dual scattering phenomenon, explain the results in Fig. 1, outline the conditions to maximize odd Willis coupling, and explore the associated exotic scattering features, we model the wave interactions with the scatterer using coupled mode equations. Consistent with the previous description, we generally model the scatterer as an open cavity supporting monopolar and a dipolar modes, with complex amplitudes ${a}_{M}$ and ${a}_{D}$ generally resonating at frequencies ${\omega }_{M}$ and ${\omega }_{D}$, with radiative decay rates ${\gamma }_{M}$ and ${\gamma }_{D}$. For simplicity, we neglect material loss for the moment. The equations of motion generally become (Supplementary Information 4–11)

3 $$\frac{d}{dt}\left(\begin{array}{c}{a}_{M}\\ {a}_{D}\end{array}\right)	=\left(\begin{array}{cc}-i{\omega }_{M}-{\gamma }_{M}-i({\beta }_{L}+{\beta }_{R})X & -F{U}_{0}+i({\beta }_{L}-{\beta }_{R})\sqrt{{\gamma }_{D}/{\gamma }_{M}}X\\ F{U}_{0}+i({\beta }_{L}-{\beta }_{R})\sqrt{{\gamma }_{D}/{\gamma }_{M}}X & -i{\omega }_{D}-{\gamma }_{D}-i({\beta }_{L}+{\beta }_{R}){\gamma }_{D}/{\gamma }_{M}X\end{array}\right)\left(\begin{array}{c}{a}_{M}\\ {a}_{D}\end{array}\right)+\\ \,	\quad+\left(\begin{array}{cc}\sqrt{2{\gamma }_{M}} & 0\\ 0 & i\sqrt{2{\gamma }_{D}}\end{array}\right)\left(\begin{array}{c}{S}_{+M}\\ {S}_{+D}\end{array}\right)\\ \,\left(\begin{array}{c}{S}_{-M}\\ {S}_{-D}\end{array}\right)	=\left(\begin{array}{cc}-1 & 0\\ 0 & -1\end{array}\right)\left(\begin{array}{c}{S}_{+M}\\ {S}_{+D}\end{array}\right)+\left(\begin{array}{cc}\sqrt{2{\gamma }_{M}} & 0\\ 0 & -i\sqrt{2{\gamma }_{D}}\end{array}\right)\left(\begin{array}{c}{a}_{M}\\ {a}_{D}\end{array}\right).$$

Here ${S}_{+M}$, ${S}_{+D}$ are the incoming scattering harmonics for monopole and dipole excitation, corresponding to the left and right column of Fig. 1a, c, e, and ${S}_{-M}$, ${S}_{-D}$ are the corresponding outgoing monopolar and dipolar scattered waves. The two modes are coupled through the off-diagonal elements in the first matrix of Eq. (3), which consist of an odd real term $F{U}_{0}$ describing the biasing flow, where ${U}_{0}$ is the rotational flow velocity and *F* is a geometrical factor, and an even imaginary term, describing the asymmetry in sound speeds in the right [${c}_{0}(1+{\beta }_{R})$] and left [${c}_{0}(1+{\beta }_{L})$] cavities, with *X* being another geometrical factor. Here ${c}_{0}$ is the unmodified sound speed and ${\beta }_{L}$, ${\beta }_{R}$ indicate the perturbation of the sound speed in the left and right cavities, respectively. A detailed derivation of (3), including explicit expressions for $F$ and $X$ as a function of the geometrical parameters of the scatterer in Fig. 1, is provided in Supplementary Information 4–11. The dual nature of the odd and even Willis terms in the off-diagonal elements is consistent with their opposite symmetry with respect to time reversal and the wave number, reflected also in their real/imaginary nature in the absence of absorption, stemming from power conservation.

We stress the importance of the third cavity in our design, which breaks mirror symmetry in the *y* direction. This is a necessary feature to have $F\;\ne\; 0$, and correspondingly the insurgence of a non-zero odd Willis coupling, as rigorously proven in Supplementary Information 8. It follows that breaking reciprocity is not sufficient to enable odd Willis coupling: removing this third cavity would yield $F=0$, hence Willis coupling would always be zero independent of the bias flow (Supplementary Information 8).

A general scatterer can support a combination of even and odd components to the Willis polarizability. A purely odd Willis scatterer, as in Fig. 1e, has ${\beta }_{L}={\beta }_{R}=0$ and Eq. (3) yield Willis coefficients (Supplementary Information 10)

4 $${\alpha }_{x}^{pv}={\alpha }_{x}^{vp}=\frac{8}{{\omega }^{2}}\frac{F{U}_{0}\sqrt{{\gamma }_{M}{\gamma }_{D}}}{{(F{U}_{0})}^{2}-(\omega -{\omega }_{M}+i{\gamma }_{M})(\omega -{\omega }_{D}+i{\gamma }_{D})}.$$

Equation (4) shows that maximum odd Willis coupling is achieved with minimum flow speed when the monopole and dipole resonances are aligned, i.e., for ${\omega }_{M}={\omega }_{D}$, as in the case of Fig. 1 since the two resonances are established within the same cavity. In this scenario, at resonance we obtain

5 $${\alpha }_{x}^{pv}={\alpha }_{x}^{vp}=\frac{8}{{\omega }^{2}}\frac{F{U}_{0}\sqrt{{\gamma }_{M}{\gamma }_{D}}}{{(F{U}_{0})}^{2}+{\gamma }_{M}{\gamma }_{D}},$$

which ensures maximum odd Willis coupling for the rotational velocity ${U}_{0}=\sqrt{{\gamma }_{M}{\gamma }_{D}}/F$, a form of critical coupling between the radiative loss of the system and the angular momentum bias that breaks time reversal. Under this condition, ${\alpha }_{x}^{vp}={\alpha }_{x}^{pv}=4{\omega }^{-2}$, as derived before. Maximum odd Willis coupling is achieved for moderate bias flows when the scatterers have a large resonance Q-factor, i.e., low radiative damping $\sqrt{{\gamma }_{M}{\gamma }_{D}}$, and/or for a large asymmetry along *y*, which ensures large *F*. Figure 1 compares in every panel the prediction stemming from our analytical model to full-wave finite-element method (FEM) simulations^33^, showing excellent agreement without the need of any fitting parameter. The optimal bias flow was chosen using Eq. (4), indeed yielding odd Willis coupling coefficients that reach the maximum value allowed for a passive scatterer.

### Experimental verification

**Fig. 2:**
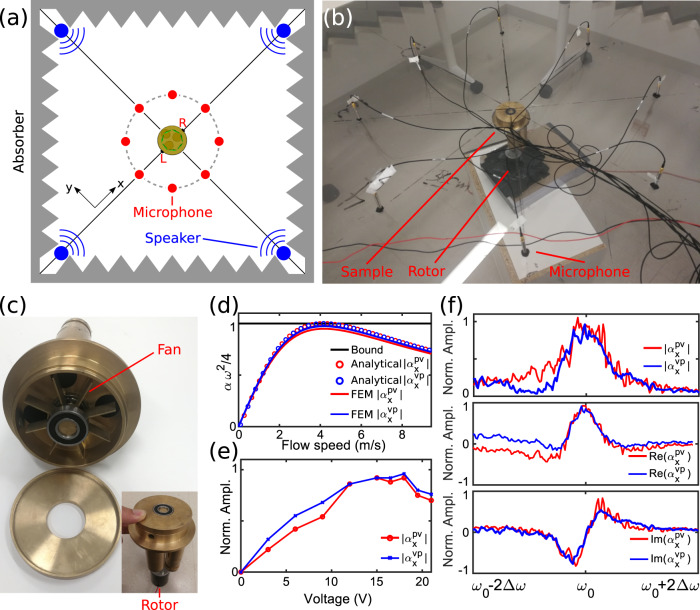
Experimental measurement of odd Willis coupling. **a**, **b** Experimental set-up. **c** Fabricated sample. **d** Analytical and numerical calculations of the Willis coefficients as a function of rotational flow speed. **e** Measured Willis coefficients as a function of applied motor voltage. **f** Measured frequency dispersion of the Willis coefficients for a motor voltage of 16.5 V.

In order to experimentally implement a scatterer with purely odd Willis coupling, we implemented the geometry in Fig. 1e in the form of a high-Q subwavelength brass resonator, as shown in Fig. 2c. A shaft connected to a rotor controls the rotational flow inside its circular cavity, and we measured the acoustic scattering in the setup shown in Fig. 2a, b, with more details provided in the Methods Section. Figure 2d shows the analytically calculated magnitude of the Willis coefficients $|{\alpha }_{x}^{pv}|$, $|{\alpha }_{x}^{vp}|$, as a function of the applied bias flow speed, according to Eq. (5), and compares it with FEM simulations. For zero bias, the magnitude of both cross-coupling polarizabilities are expected to be zero, because the scatterer is mirror and time-reversal symmetric. As the bias flow increases, however, T-symmetry is broken and the odd Willis coefficients increase, reaching the bound for *U*_0_ = 4.1 m/s. Beyond this point, the cross-coupling polarizabilities decrease.

In our experiments, we collected the scattered sound around the object, and retrieved ${\alpha }_{x}^{pv}$ and ${\alpha }_{x}^{vp}$ as a function of the applied voltage on the motor, whose magnitudes are shown in Fig. 2e, indeed following a similar trend. For an applied voltage around 16.5 V, the cross-coupling polarizabilities reach their maximum, consistent with the numerical results in Fig. 2d. Figure 2f presents the frequency dispersion of the measured cross-coupling polarizabilities for this applied motor voltage, showing a profile consistent with our analytical and numerical results in Fig. 1f, and confirming the odd-symmetric nature of the observed Willis coupling. The curves do not precisely overlap with each other, as a symptom of small imperfections in our practical implementation, which introduce unwanted geometrical asymmetries, and also due to the presence of the fan blades that are not accounted in our model. However, overall they confirm the observation of odd-symmetric Willis coupling in a non-reciprocal acoustic scatterer.

### Geometrical and loss asymmetries

**Fig. 3:**
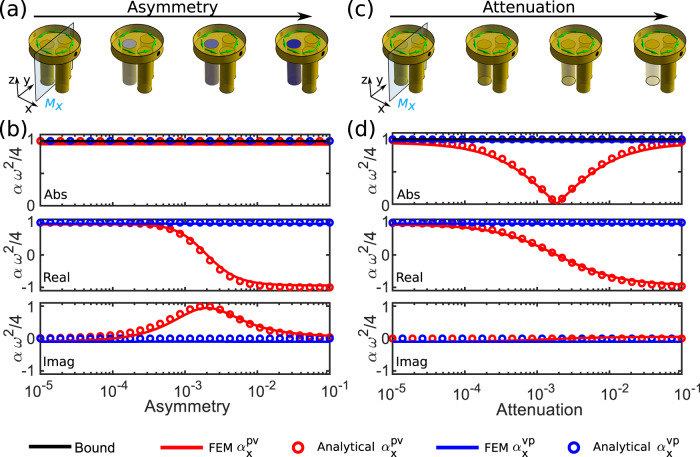
Role of geometrical asymmetries and loss. **a**, **b** Evolution of the Willis coefficients as a function of geometrical asymmetry in the left cavity. **c**, **d** Evolution of the Willis coefficients as a function of loss in the left cavity.

Our analytical model can efficiently capture the effects of geometrical asymmetries and loss in the system, responsible for the deviations between experiments and calculations. If both mirror and time-reversal symmetries are generally broken, the Willis coupling is split into its odd and even parts, i.e., ${\alpha }_{x}^{pv}={\alpha }^{o}-{\alpha }^{e}$ and ${\alpha }_{x}^{vp}={\alpha }^{o}+{\alpha }^{e}$. Consider for instance the case of a denser material loading the left cylindrical cavity in the scatterer of Fig. 1a, with modified sound speed ${c}_{0}(1+{\beta }_{L})$, while the right cavity has sound speed ${c}_{0}$, as in Fig. 3a. At the resonance frequency $\omega ={\omega }_{M}={\omega }_{D}$, by solving Eq. (3) we obtain (Supplementary Information 11)

6 $${\alpha }^{o}=\frac{8}{{\omega }^{2}}\frac{F{U}_{0}\sqrt{{\gamma }_{D}{\gamma }_{M}}}{{(F{U}_{0})}^{2}+i2{\beta }_{L}X{\gamma }_{D}+{\gamma }_{M}{\gamma }_{D}},\,{\alpha }^{e}=\frac{8}{{\omega }^{2}}\frac{i{\beta }_{L}X{\gamma }_{D}}{{(F{U}_{0})}^{2}+i2{\beta }_{L}X{\gamma }_{D}+{\gamma }_{M}{\gamma }_{D}}.$$

As expected, odd Willis coupling is proportional to the rotational flow velocity ${U}_{0}$, while the even coupling is proportional to the geometrical asymmetry ${\beta }_{L}$. At the optimal flow speed ${U}_{0}=\sqrt{{\gamma }_{M}{\gamma }_{D}}/F$, Eq. (3) yields

7 $${\alpha }_{x}^{vp}=\frac{4}{{\omega }^{2}},\,{\alpha }_{x}^{pv}=\frac{4}{{\omega }^{2}}\frac{{\gamma }_{M}-i{\beta }_{L}X}{{\gamma }_{M}+i{\beta }_{L}X},$$

indicating that at resonance the magnitude of the Willis coefficients is still equal and maximum, but their phase is no longer identical. Figure 3b shows the Willis coefficients as a function of ${\beta }_{L}$, using Eq. (7) and comparing it to full-wave FEM simulations. Indeed, we find ${\alpha }_{x}^{vp}=4{\omega }^{-2}$ independent of the geometrical asymmetry, while the phase of ${\alpha }_{x}^{pv}$ varies from 0 to $\pi$ as ${\beta }_{L}X$ increases. As expected, for very large geometrical asymmetries, the Willis coupling is overall even in nature, as the geometrical asymmetry dominates, flipping sign. By considering the opposite asymmetry, i.e., introducing a denser medium in the right cavity, we obtain the dual response: constant ${\alpha }_{x}^{pv}=4{\omega }^{-2}$, and a phase variation for ${\alpha }_{x}^{vp}$.

This degree of control of the two Willis coefficients through the asymmetry in the two cavities at the resonance of our optimized structure is explained by inspecting the field profile at the optimal velocity flow, shown in Fig. 1e: the interference of monopolar and dipolar fields in the scatterer ensures that, for pressure excitation, the left cavity has zero fields, while for velocity excitation the right cavity is not excited. This result, proven analytically in Supplementary Information 12, indeed explains why for a left (right) perturbation ${\alpha }_{x}^{vp}$ (${\alpha }_{x}^{pv}$) is not affected, while the other coefficient changes phase. Interestingly, if we reverse the air flow, we reverse the field distributions, and hence the overall effect is reversed.

So far, we have shown full control of the phase of the Willis coefficients through geometrical asymmetries and bias flow, but not of their magnitude, consistent with energy conservation. In Supplementary Information 3, we indeed prove that $|{\alpha }_{x}^{vp}|=|{\alpha }_{x}^{pv}|$ applies generally to any lossless scatterer. The effect of loss can be also incorporated and modeled in our formulation. An asymmetric loss distribution can be modeled through an imaginary value of ${\beta }_{L}=-i{\beta^ {\prime\prime} }_{L}$ in (7). In this scenario, we find in Fig. 3d that ${\alpha }_{x}^{vp}$ is still not affected by loss, as expected for the previous considerations, while $|{\alpha }_{x}^{pv}|$ decreases and becomes identically zero for ${\beta ^{\prime\prime} }_{L}={\gamma }_{M}/X$. This scenario implies a scatterer with extreme non-reciprocal features, for which a pressure field excites maximum dipolar scattering, but the velocity field does not excite any monopole. This operation can only arise in non-reciprocal and lossy scatterers at the critical coupling condition.

### Applications

Bianisotropic scatterers based on geometrical asymmetries have been opening exciting opportunities in several scattering problems in optics and electromagnetics, including asymmetric absorption^23,24^, topological order^25^, and wavefront engineering^26^, recently extended also to acoustics in the context of even Willis coupling^21,27–30,34^. The dual form of Willis coupling introduced in this paper opens opportunities in this context, adding non-reciprocity as a knob, with important implications for scattering theory and applications. For instance, topological acoustics with non-reciprocal features based on angular momentum bias has been envisioned in^35,36^, and the introduced concepts can enhance these phenomena and achieve topologically robust transport and one-way flow of sound in optimal ways.

**Fig. 4:**
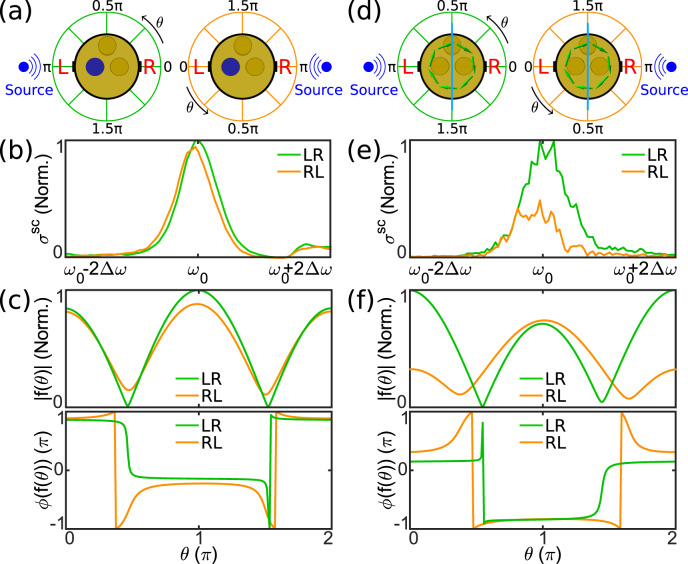
Sound scattering and Willis coupling. **a** Even Willis scatterer excited by an incident wave from the left (green case) and right (orange case). **b** Measured total scattering width for the even Willis scatterer for an incident wave from the left (green line) and right (orange line). **c** Measured angular dependence of the pressure field scattered by an even Willis scatterer in magntiude (top) and phase (bottom). **d**, **e**, **f** Similar to **a**, **b**, **c** for the odd Willis scatterer.

As a striking example of the counterintuitive scattering phenomena enabled by odd Willis coefficients, Fig. 4 compares the total scattering width and the scattering pattern measured for plane waves propagating from the left (green curves) and right (orange curves), and exciting our even (left panels) and odd (right panels) Willis scatterers. An even Willis scatterer, despite being geometrically asymmetric, is bound by reciprocity to support identical forward scattering when excited from specular directions. Because of the optical theorem^37^, this property translates into the fact that reciprocal scatterers, despite their arbitrary geometrical asymmetry, have always the same total scattering width for opposite excitations, i.e., the total scattered power when excited from opposite directions must be the same. We have experimentally verified these features in Fig. 4b, c, which indeed show how, for every frequency the total scattering width (Supplementary Information 14) is equal for our even Willis scatterer (Fig. 4b, apart from small deviations due to our measurements). Similarly, Fig. 4c shows that the angular dependence $f(\theta )$ of the scattered pressure fields at the resonance frequency ${\omega }_{0}$, is generally different for opposite excitations, but the forward scattering $f\left(0\right)$ is equal because of reciprocity. In particular, despite the fact that the backward scattering $f\left(\pi \right)$, and correspondingly the reflected power, is generally different for an asymmetric scatterer, the integrated power over all angles is necessarily the same. These measurements are validated by our full-wave simulations in Supplementary Fig. S3, and similar considerations apply to any asymmetric scatterer obeying reciprocity.

Figure 4d considers the dual scenario of our odd Willis scatterer. In this case, interestingly we observe the dual scattering response: the backward scattering $f(\pi )$ and the reflected power are necessarily identical for a purely odd Willis scatterer, but the forward scattering $f\left(0\right)$ can vary widely as a consequence of broken reciprocity (Fig. 4f). In turn, because of the optical theorem this property implies that odd Willis scatterers support largely asymmetric total scattering widths and scattered power levels for opposite excitations, as experimentally verified in Fig. 4e. These features highlight the dual scattering properties of odd Willis scatterers, with exciting potential for scattering manipulation and sound control.

When combined in arrays, these features also open opportunities for metasurfaces. For instance, a periodic array of odd Willis scatterers can realize a non-reciprocal meta-grating for efficient circulation of airborne sound, as recently envisioned in electromagnetics in ref. ^38^. Meta-gratings have been recently introduced to overcome the limitations of gradient metasurfaces and enable wavefront steering towards extreme angles with unitary efficiency^21,26^. This concept, originally discussed in electromagnetics and relying on bianisotropy, has been recently extended to acoustics based on suitably tailored geometrically asymmetric scatterers with even Willis coefficients^21,39,40^. These meta-gratings inherently obey reciprocity, i.e., their scattering matrix is necessarily symmetric, and if efficient wave steering is achieved from normal incidence to grazing angle, a wave impinging from the grazing direction necessarily is routed back towards normal incidence. A meta-grating formed by odd Willis scatterers with optimal air flow as in Fig. 1e, on the contrary, can realize efficient wavefront steering with non-reciprocal features.

**Fig. 5:**
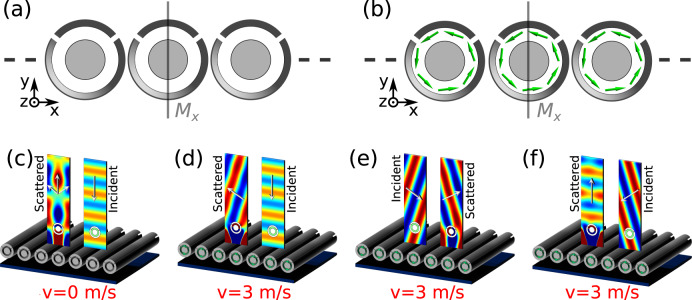
Non-reciprocal meta-grating based on odd Willis inclusions. **a**, **b** An acoustic grating with geometrically symmetric unit cells. **c** With no bias flow, for a normally incident plane wave the reflected power is steered symmetrically towards left and right Floquet channels. **d** For a bias velocity of 3 m/s, a normally incident wave is totally reflected into the -1 diffraction order. **e** For excitation from the -1 diffraction direction, instead, all energy is routed towards the +1 channel, confirming large non-reciprocity and isolation for free-space airborne sound waves. **f** For excitation from the +1 order, unitary reflection is achieved towards normal incidence.

In Fig. 5a, we consider an array of mirror-symmetric scatterers with radius of 4 cm and a period of 15 cm, larger than the wavelength so that the ±1 diffraction orders are supported for illumination at normal incidence. As shown in Fig. 5c, in the absence of bias flow in the scatterers, and hence no Willis coupling, all reflected energy is distributed symmetrically with respect to the normal. We next apply a modest bias flow, with velocity of 3 m/s, in the scatterers, as shown in Fig. 5b. Maximum odd Willis coupling is induced, and all incident energy is redirected to the −1 diffraction order (Fig. 5d). If we now excite the array with a wave incident from the -1 order, the reflected wave is not returned to the normal direction, but it is instead re-routed to the +1 channel (Fig. 5e), confirming large non-reciprocal response and isolation for airborne sound waves in free space. Figure 5f shows the case of excitation from the right side, which is now fully reflected towards the normal, creating an ideal free-space circulator for sound waves.

## Discussion

To conclude, in this paper, we have introduced and experimentally observed a dual form of Willis coupling arising in an acoustic scatterer whose geometry obeys mirror symmetry along the direction of propagation, inherently odd-symmetric in nature with respect to time-reversal, and discussed the opportunities that it enables in the context of sound control and manipulation. Through a rotational flow bias that breaks time-reversal symmetry combined with the suitable design of a resonant cavity, we have induced a dual form of coupling between pressure and velocity for sound, inherently odd-symmetric in nature, which provides highly exotic sound-matter interactions when suitably enhanced through resonant excitations. We derived the conditions to enable this effect, which requires broken symmetry in the plane transverse to the direction of propagation, derived the optimal bias flow velocity that maximizes its effect, and demonstrated its application in scattering phenomena, such as the demonstration of largely asymmetric total scattering widths for opposite excitation, and next-generation acoustic meta-gratings supporting circulation for airborne sound and non-reciprocal wavefront steering with unitary efficiency. Our results pave the way towards opportunities for sound control and acoustic engineering based on Willis phenomena enabled by time-reversal symmetry breaking, with exciting opportunities for bio-medical imaging, sonar technology, and wavefront engineering, such as exotic asymmetric absorption, transmission, scattering and topological wave phenomena. More broadly, similar concepts can be envisioned in elasto-dynamics, mechanics and even optics and photonics, for instance based on suitably tailored magnetically biased nanoparticles, or spatio-temporal modulation, opening a plethora of directions for metamaterial technology and applications.

## Methods

### Full-wave simulations

The full-wave numerical simulations in the main text were performed using the Pressure Acoustic module coupled with Linearized Potential Flow module of the commercialized finite-element software COMSOL Multiphysics 5.4, assuming a mathematically imposed, time-independent bias velocity as the background flow inside the cavity. The walls of the sample were set as rigid wall as boundary conditions. The simulation was performed in the frequency domain for background incident field coming from *x*-, -*x*-, *y*-, and -*y*-directions, respectively. The polarizabilities retrieval is performed according to Ref. ^21^.

### Experimental set-up

The sample was machined with brass through a CNC machine. The outer radius of the sample is 40 mm. The thickness of the wall is 4 mm and hence the inner radius of the cavity is 36 mm. The height of the cavity is 20 mm. The inner radius of each 3 cylinder is 9 mm. The height of the side cylinder is 74.3 mm. The total height of the middle cylinder is 90 mm with an adjustment screw that can change its height. The height of the screw is 28 mm. All three cylinders’ centers are located in the radius of 27 mm to the center. Two PGN-R8-2RS sealed ball bearing with standard size 1/2″ × 1–1/8″ × 5/16″ is placed on the top and bottom of the cavity with a shaft through them. The shaft is connected to a DC motor with maximum voltage 24 V. The cavity is connected to the outside with two holes above the side cylinders. The radius of the hole is 4 mm, and the holes are 10 mm above the side cylinders. In the experiment, the sample was placed in a two-dimensional planar Acrylic sheet waveguide with 1.83 m long by 1.83 m wide, with height of 2.5 cm. Sound absorbing materials were placed at the boundaries of the waveguide to ensure a free acoustic field environment. Eight microphones were placed 38.1 cm away from the center of the scatter to measure the scattered fields.

### Measurement and data processing

The acoustic pressure was measured using 8 B&K 1/8″ microphones. We placed 4 loudspeakers in each of 4 corners. During measurement, we activate each loudspeaker one by one to mimic incident waves coming from *x*-, -*x*-, *y*-, and -*y*-directions, respectively. We first measured the background field without the sample. Then we placed the sample back to measure the total field. The scattered field is calculated using total field minus the background field. We align the monopole resonance and dipole resonance by adjust the screw and adding water into the cylinders. For the measurement performed in the paper, the screw is moved 2.5 mm away from its top with 8 mL water injected into the middle cylinder and 10 mL water injected into both side cavities. In real measurement, the measured centered frequency is 1560 Hz, with $\varDelta f=80\,Hz$. In data processing, we normalized the polarizabilities by 5 × 10^−9^. We estimate that the peak polarizability value in our measurements was about 15% of the maximum possible value achievable in a lossless, ideal scatterer, due to the presence of unwanted imperfections, material loss, and noise caused by the motor.

## Supplementary information

Supplementary Information

## Data Availability

All relevant data that support the findings of this study are available from the corresponding author upon reasonable request.
