# Supplementary material for: Odd Willis coupling induced by broken time-reversal symmetry
Source: Nat Commun. 2021 May 10;12:2615. doi: 10.1038/s41467-021-22745-5 (PMC8110991; doi:10.1038/s41467-021-22745-5)
Supplement: Supplementary file 1 — Supplementary Information [file 41467_2021_22745_MOESM1_ESM.pdf]

Supplementary Information

Odd Willis Coupling Induced by Broken Time-Reversal Symmetry

Li Quan[1], Simon Yves[2], Yugui Peng[2], Hussein Esfahlani[2] and Andrea Alù[1,2,3,*]

[1]*Department of Electrical and Computer Engineering, The University of Texas at Austin, Austin, Texas 78712, USA*

[2]*Photonics Initiative, Advanced Science Research Center, City University of New York, New York, NY 10031, USA*

[3]*Physics Program, Graduate Center, City University of New York, New York, NY 10026, USA*

[*]To whom correspondence should be addressed (email: aalu@gc.cuny.edu)

Contents

1. Unit system 2
2. Bound for passive Willis scatterers 2
3. Proof of relation $\left|\alpha_x^{vp}\right| = \left|\alpha_x^{pv}\right|$ when the scatterer is passive and lossless 4
4. Derivation of the modal solutions in the scatterer 5
5. Eigen-solutions 8
   5.1 Anti-symmetric (dipole) mode 9
   5.2 Symmetric (monopole) mode 10
6. Normalization 11
7. Eigen-solution in the presence of bias 12
8. Symmetry considerations 14
9. S-matrix and polarizability tensor 17
10. Scattering analysis 20
11. Role of asymmetries and loss 23
12. Location of the pressure node in Fig. 1e 26
13. Duality in scattering 27
14. Scattering pattern and total scattering width 28

1. Unit system

In the main paper, we use normalized units following the notation in Ref. 21 in the main paper, in order to simplify the notation throughout the paper. The conversion to SI units is summarized in Table 1.

Table 1. Conversion between SI units and the unit system used in our paper

Quantity	Symbol	Relation to SI
Monopole	M	$-\sqrt{2}M_{SI}$
Dipole	$\mathbf{D}$	$ik_0\mathbf{D}_{SI}$
Pressure	p	$\frac{p_{SI}}{\sqrt{2}}$
Velocity	$\mathbf{v}$	$\rho_0 c_0 \mathbf{v}_{SI}$
Pressure-Pressure Polarizability	α^{pp}	$-2\alpha_{SI}^{pp}$
Velocity-Velocity Polarizability	$\overleftrightarrow{\boldsymbol{\alpha}}^{vv}$	$\frac{ik_0\overleftrightarrow{\boldsymbol{\alpha}}_{SI}^{vv}}{\rho_0 c_0}$
Pressure-Velocity Polarizability	$\overleftrightarrow{\boldsymbol{\alpha}}^{pv}$	$-\frac{\sqrt{2}\overleftrightarrow{\boldsymbol{\alpha}}_{SI}^{pv}}{\rho_0 c_0}$
Velocity-Pressure Polarizability	$\overleftrightarrow{\boldsymbol{\alpha}}^{vp}$	$ik_0\sqrt{2}\overleftrightarrow{\boldsymbol{\alpha}}_{SI}^{vp}$

2. Bound for passive Willis scatterers

For passive Willis scatterers, energy conservation requires the following inequality to be satisfied[21]:

$$\mathrm{Diag}\left[\omega^2\left(\overleftrightarrow{\boldsymbol{\alpha}}^{*T}\overleftrightarrow{\boldsymbol{\alpha}}\right)\right] \le \mathrm{Diag}\left[4i\left(\overleftrightarrow{\boldsymbol{\alpha}}^{T*}-\overleftrightarrow{\boldsymbol{\alpha}}\right)\right]. \quad \text{(S1)}$$

In the two-dimensional scenario, consistent with the geometry in the main paper, the polarizability tensor is expressed as

$$\overleftrightarrow{\boldsymbol{\alpha}} = \begin{pmatrix} \alpha^{pp} & \alpha_x^{pv} & \alpha_y^{pv} \\ \alpha_x^{vp} & \alpha_{xx}^{vv} & \alpha_{xy}^{vv} \\ \alpha_y^{vp} & \alpha_{yx}^{vv} & \alpha_{yy}^{vv} \end{pmatrix} \tag{S2}$$

By replacing Eq. (S2) into Eq. (S1), we get:

$$\begin{cases} \sqrt{\left|\alpha_x^{vp}\right|^2 + \left|\alpha_y^{vp}\right|^2} \le \sqrt{\dfrac{-\dfrac{8}{\omega^2}\operatorname{Im}\left(1/\alpha^{pp}\right) - 1}{\left|1/\alpha^{pp}\right|^2}} \le \dfrac{4}{\omega^2} \\ \sqrt{\left|\alpha_x^{pv}\right|^2 + \left|\alpha_{yx}^{vv}\right|^2} \le \sqrt{\dfrac{-\dfrac{8}{\omega^2}\operatorname{Im}\left(1/\alpha_{xx}^{vv}\right) - 1}{\left|1/\alpha_{xx}^{vv}\right|^2}} \le \dfrac{4}{\omega^2} \\ \sqrt{\left|\alpha_y^{pv}\right|^2 + \left|\alpha_{xy}^{vv}\right|^2} \le \sqrt{\dfrac{-\dfrac{8}{\omega^2}\operatorname{Im}\left(1/\alpha_{yy}^{vv}\right) - 1}{\left|1/\alpha_{yy}^{vv}\right|^2}} \le \dfrac{4}{\omega^2} \end{cases} \tag{S3}$$

If the scatterer is subwavelength, and only resonant in the *x*-direction, we have $\alpha_y^{vp} \approx 0$, $\alpha_{yx}^{vv} \approx 0$.

By replacing these relations into Eq. (S3), we get the maximum bound for passive Willis scatterers:

$$\begin{cases} \left|\alpha_x^{vp}\right| \le \sqrt{\dfrac{-\dfrac{8}{\omega^2}\operatorname{Im}\left(1/\alpha^{pp}\right) - 1}{\left|1/\alpha^{pp}\right|^2}} \le \dfrac{4}{\omega^2} \\ \left|\alpha_x^{pv}\right| \le \sqrt{\dfrac{-\dfrac{8}{\omega^2}\operatorname{Im}\left(1/\alpha_{xx}^{vv}\right) - 1}{\left|1/\alpha_{xx}^{vv}\right|^2}} \le \dfrac{4}{\omega^2} \end{cases} . \tag{S4}$$

Notice that this bound is not relying on reciprocity, hence it applies to arbitrary linear, lossless and /or lossy scatterers. These can be generalized to 3D scatterers and to multiple resonances in different directions, following a similar derivation. The equality in Eqs. (S4) can be achieved at resonance in suitably optimized lossless scatterers.

3. Proof of relation $\left|\alpha_x^{vp}\right| = \left|\alpha_x^{pv}\right|$ when the scatterer is passive and lossless

For passive and lossless scatterers, energy conservation requires[21]

$$\mathrm{Diag}\left[\omega^2\left(\vec{\vec{\boldsymbol{\alpha}}}^{*T}\vec{\vec{\boldsymbol{\alpha}}}\right)\right] \le \mathrm{Diag}\left[4i\left(\vec{\vec{\boldsymbol{\alpha}}}^{T*}-\vec{\vec{\boldsymbol{\alpha}}}\right)\right]. \tag{S5}$$

Again, assuming only a resonance in the *x*-direction, the condition can be simplified as

$$\left(\omega^2\alpha^{pp}-4i\right)\alpha_x^{pv*}+\left(\omega^2\alpha_{xx}^{vv}-4i\right)^*\alpha_x^{vp}=0, \tag{S6}$$

which can be written as

$$-\frac{\alpha_x^{vp}}{\alpha_x^{pv*}}=\frac{\left(\alpha^{pp}-\dfrac{4i}{\omega^2}\right)}{\left(\alpha_{xx}^{vv}-\dfrac{4i}{\omega^2}\right)^*}. \tag{S7}$$

By taking the conjugate of Eq. (S7), we have

$$-\frac{\alpha_x^{vp*}}{\alpha_x^{pv}}=\frac{\left(\alpha^{pp}-\dfrac{4i}{\omega^2}\right)^*}{\left(\alpha_{xx}^{vv}-\dfrac{4i}{\omega^2}\right)}. \tag{S8}$$

Multiplying Eq. (S7) with (S8), we get

$$\left|\frac{\alpha_x^{vp}}{\alpha_x^{pv*}}\right|^2=\frac{\left(\alpha^{pp}-\dfrac{4i}{\omega^2}\right)\left(\alpha^{pp*}+\dfrac{4i}{\omega^2}\right)}{\left(\alpha_{xx}^{vv}-\dfrac{4i}{\omega^2}\right)\left(\alpha_{xx}^{vv*}+\dfrac{4i}{\omega^2}\right)}=\frac{\left|\alpha^{pp}\right|^2-\dfrac{8}{\omega^2}\mathrm{Im}\left(\alpha^{pp}\right)+\dfrac{16}{\omega^4}}{\left|\alpha_{xx}^{vv}\right|^2-\dfrac{8}{\omega^2}\mathrm{Im}\left(\alpha_{xx}^{vv}\right)+\dfrac{16}{\omega^4}}. \tag{S9}$$

For lossless scatterers, Eqs. (S4) take the equal sign and can be rewritten as

$$\left|\alpha_x^{vp}\right|^2=\frac{-\dfrac{8}{\omega^2}\mathrm{Im}\left(1/\alpha^{pp}\right)-1}{\left|1/\alpha^{pp}\right|^2}=\frac{8}{\omega^2}\mathrm{Im}\left(\alpha^{pp}\right)-\left|\alpha^{pp}\right|^2 \tag{S10}$$

$$\left|\alpha_x^{pv}\right|^2=\frac{-\dfrac{8}{\omega^2}\mathrm{Im}\left(1/\alpha_{xx}^{vv}\right)-1}{\left|1/\alpha_{xx}^{vv}\right|^2}=\frac{8}{\omega^2}\mathrm{Im}\left(\alpha_{xx}^{vv}\right)-\left|\alpha_{xx}^{vv}\right|^2. \tag{S11}$$

By replacing Eqs. (S10) and (S11) into the right part of Eq. (S9), we get

$$\frac{\left|\alpha_x^{vp}\right|^2}{\left|\alpha_x^{pv}\right|^2}=\frac{\left|\alpha^{pp}\right|^2-\frac{8}{\omega^2}\operatorname{Im}\left(\alpha^{pp}\right)+\frac{16}{\omega^4}}{\left|\alpha_{xx}^{vv}\right|^2-\frac{8}{\omega^2}\operatorname{Im}\left(\alpha_{xx}^{vv}\right)+\frac{16}{\omega^4}}=\frac{-\left|\alpha_x^{vp}\right|^2+\frac{16}{\omega^4}}{-\left|\alpha_x^{pv}\right|^2+\frac{16}{\omega^4}}. \tag{S12}$$

Simplifying Eq. (S12) we obtain the condition $\left|\alpha_x^{vp}\right|=\left|\alpha_x^{pv}\right|$.

4. Derivation of the modal solutions in the scatterer

In the derivation of the wave equation, for convenience we use the conventional SI unit system. The mass conservation and momentum equations for sound in the cavity can be expressed as

$$\frac{\partial\rho}{\partial t}+\nabla\cdot\left(\rho\vec{u}\right)+\frac{\rho u_2}{h_w}=0 \tag{S13}$$

$$\rho\frac{d\vec{u}}{dt}+\nabla P=0. \tag{S14}$$

Here ρ, $\vec{u}$, P represent the density, particle velocity and pressure, respectively, u_2 is the mass contribution of the cylinder to the mass conservation equation, h_w is the height of the cavity in the *z*-direction. We decompose the density, particle velocity and pressure into biased and acoustic parts as:

$$\rho=\rho_0+\rho' \tag{S15}$$

$$\vec{u}=\vec{U}_0+\vec{v} \tag{S16}$$

$$P=P_0+p \tag{S17}$$

The coordinate system is shown in Fig. S1. We assume rotational flow in the cavity as

$$\vec{U}_0 = \Omega r \hat{\theta} . \tag{S18}$$

By replacing Eqs. (S15), (S16), (S17) and (S18) into Eqs. (S13) and (S14), and considering $\frac{d}{dt} = \frac{\partial}{\partial t} + \vec{u} \cdot \nabla$, we obtain the linearized mass conservation equation and the momentum equation in $\hat{r}$ and $\hat{\theta}$ directions, respectively:

$$\frac{\partial \rho'}{\partial t} + \rho_0 \frac{1}{r} \frac{\partial (r v_r)}{\partial r} + \rho_0 \frac{\partial v_\theta}{r \partial \theta} + \Omega \frac{\partial \rho'}{\partial \theta} + \frac{\rho_0 u_2}{h_w} = 0 \tag{S19}$$

$$\hat{r} \left[\rho_0 \left(-i\omega v_r + \Omega \frac{\partial v_r}{\partial \theta} - 2\Omega v_\theta \right) - \rho' \Omega^2 r + \frac{\partial p}{\partial r} \right] = 0 \tag{S20}$$

$$\hat{\theta} \left[\rho_0 \left(-i\omega v_\theta + 2\Omega v_r \hat{\theta} + \hat{\theta} \Omega \frac{\partial v_\theta}{\partial \theta} \right) + \frac{\partial p}{r \partial \theta} \right] = 0 . \tag{S21}$$

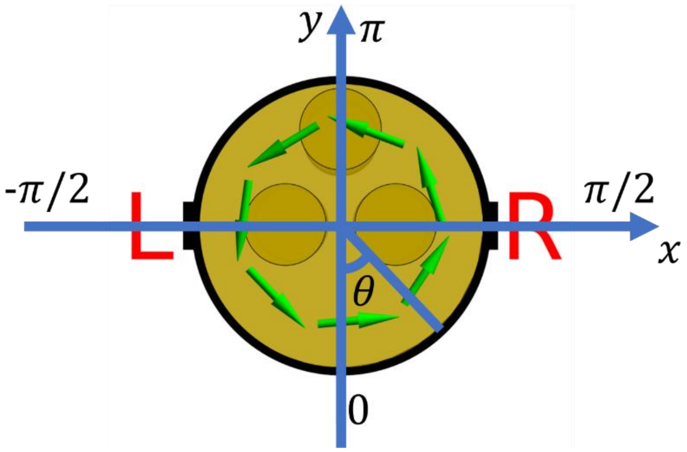

Fig. S1. Reference system and geometry of the scatterer under analysis.

We suppose that the inner and outer radii of the cavity are very close to each other, and hence $v_r = 0$, hence we only need to consider the momentum equation in the $\hat{\theta}$ direction. By considering

the constitutive and impedance relations

$$\rho' = \frac{p}{c_0^2} \tag{S22}$$

$$u_2 = Y(\theta)\, p \tag{S23}$$

Eqs. (S19) and (S21) are then simplified as

$$i\frac{\partial p}{\partial t} = -\frac{i}{r\kappa_0}\frac{\partial v_\theta}{\partial \theta} - i\frac{Y}{h_w \kappa_0} p - i\Omega\frac{\partial p}{\partial \theta} \tag{S24}$$

$$i\frac{\partial v_\theta}{\partial t} = -\frac{i}{r\rho_0}\frac{\partial p}{\partial \theta} - i\Omega\frac{\partial v_\theta}{\partial \theta}\ . \tag{S25}$$

Here Y is the effective admittance, satisfying

$$Y = \left[Y_1''\delta(\theta-\pi) + Y_2''\delta\left(\theta+\frac{\pi}{2}\right) + Y_2''\delta\left(\theta-\frac{\pi}{2}\right) \right] \frac{\pi r_h^2}{r\Delta r} \tag{S26}$$

$$Y_1'' = \frac{-i\tan(kl_1)}{\rho_0 c_0} \tag{S27}$$

$$Y_2'' = \frac{-i\tan(kl_2)}{\rho_0 c_0} \tag{S28}$$

with r_h and Δr being the radius of the cylinder and the distance between outer and inner radius of the cavity. Y_1'' indicates the admittance of the cylinder in the middle plane with l_1 indicating the cylinder length, and Y_2'' indicates the admittance of the cylinder on the left and right sides with l_2 indicating the cylinder length. Here both Y_1'' and Y_2'' are purely imaginary values, since we assume lossless materials.

When $\Omega = 0$, we can write Eqs. (S24) and (S25) in the Hamiltonian form

$$i\frac{\partial}{\partial t}|\Psi\rangle = H_0|\Psi\rangle, \tag{S29}$$

where

$$|\Psi\rangle = \begin{pmatrix} p \\ \rho_0 c_0 v_\theta \end{pmatrix} \tag{S30}$$

and

$$H_0 = \begin{pmatrix} -i\frac{Y\rho_0 c_0^2}{h_w} & -\frac{ic_0}{r}\frac{\partial}{\partial\theta} \\ -\frac{ic_0}{r}\frac{\partial}{\partial\theta} & 0 \end{pmatrix}. \tag{S31}$$

The inner product is defined as

$$\langle\Psi_a|\Psi_b\rangle = \iiint\left[p_a^* p_b + \left(\rho_0 c_0 v_{\theta a}^*\right)\left(\rho_0 c_0 v_{\theta b}\right)\right]d\theta. \tag{S32}$$

When $\Omega \neq 0$, we can write Eqs. (S24) and (S25) as

$$\left(H_0 + \delta H\right)|\Psi\rangle = \omega|\Psi\rangle \tag{S33}$$

where

$$\delta H = \begin{pmatrix} -i\Omega\frac{\partial}{\partial\theta} & 0 \\ 0 & -i\Omega\frac{\partial}{\partial\theta} \end{pmatrix} \tag{S34}$$

5. Eigen-solutions

In the absence of bias $\Omega = 0$, we combine Eqs. (S24) and (S25), and obtain

$$\frac{\partial^2 p}{\partial \theta^2} + i\omega r^2 \frac{Y\rho_0}{h_w} p + k_0^2 r^2 p = 0 \,. \tag{S35}$$

According to Eq. (S26), the boundary condition requires

$$\left(\frac{\partial p}{\partial \theta_+} - \frac{\partial p}{\partial \theta_-} \right) + i\omega r^2 \frac{Y_n \rho_0}{h_w} p = 0 \,, \tag{S36}$$

with

$$Y_n = -i \frac{\pi r_h^2}{r \Delta r \rho_0 c_0} \tan\left(k l_n\right) \tag{S37}$$

and $n = 1, 2$. Here Y_n is again a purely imaginary number. The cavity supports two eigen-modes, as derived below.

5.1 Anti-symmetric (dipole) mode

The anti-symmetric modal solution in the cavity is given by

$$p_D = \begin{cases} A \sin\left(k_D r \theta\right) & \textit{for} \quad -\frac{\pi}{2} < \theta < \frac{\pi}{2} \\ B \sin\left(k_D r \left(\theta - \pi\right)\right) & \quad\quad\ \frac{\pi}{2} < \theta < \frac{3\pi}{2} \end{cases} \,. \tag{S38}$$

Here k_D is the eigen-wavenumber of the dipole mode, with A and B being undetermined coefficient. At $\theta = \frac{\pi}{2}$, the pressure continuity requires

$$A = -B \,. \tag{S39}$$

According to the boundary condition Eq. (S36), and considering Eq. (S39), we find that the eigen-angular frequency ω_D of the dipole mode satisfies

$$\cot\left(\omega_D \frac{r\pi}{2c_0} \right) = Y_2' \,. \tag{S40}$$

Here Y_2' is a purely real number given by

$$Y_2' = \frac{iY_2\rho_0 c_0 r}{2h_w} = \frac{\pi r_h^2}{2h_w \Delta r}\tan\left(kl_2\right). \tag{S41}$$

5.2 Symmetric (monopole) mode

The symmetric mode in the cavity has the form

$$p_M = \begin{cases} A_1 \cos\left(k_M r\theta\right) & -\frac{\pi}{2} < \theta < \frac{\pi}{2} \\ B_1 \cos\left(k_M r(\theta-\pi)\right) + B_2 \sin\left(k_M r(\theta-\pi)\right) \quad for & \frac{\pi}{2} < \theta < \pi \\ B_1 \cos\left(k_M r(\theta-\pi)\right) - B_2 \sin\left(k_M r(\theta-\pi)\right) & \pi < \theta < \frac{3\pi}{2} \end{cases} . \tag{S42}$$

Here k_M is the eigen-wavenumber of the monopole mode, with A_1, B_1, and B_2, being undetermined coefficients. At $\theta = \pi$, the boundary condition (S36) requires

$$B_2 = Y_1' B_1 . \tag{S43}$$

Here Y_1' is a purely real number, satisfying

$$Y_1' = \frac{iY_1\rho_0 c_0 r}{2h_w} = \frac{\pi r_h^2}{2h_w \Delta r}\tan\left(kl_1\right) . \tag{S44}$$

At $\theta = \frac{\pi}{2}$, the continuity of pressure requires

$$B_1 = A_1 \frac{\cot\left(k_M r \frac{\pi}{2}\right)}{\cot\left(k_M r \frac{\pi}{2}\right) - Y_1'} . \tag{S45}$$

With Eqs. (S43) and (S45), there is only one undetermined coefficient A_1 in the expression in Eq. (S42). At $\theta = \frac{\pi}{2}$, the boundary condition (S36) requires

$$B_1 + Y_1' B_1 \cot\left(k_0 r \frac{\pi}{2}\right) + A_1 + 2Y_2' A_1 \cot\left(k_0 r \frac{\pi}{2}\right) = 0 . \tag{S46}$$

By replacing Eqs. (S43) and (S45) into Eq. (S46), we obtain the eigen-angular frequency ω_M of the monopole mode as the solution of

$$\cot\left(\omega_M \frac{\pi r}{2c_0}\right) = \frac{\left(Y_1' Y_2' - 1\right) \pm \sqrt{1 + Y_1'^2 Y_2'^2 + Y_1'^2}}{Y_1' + 2Y_2'}. \tag{S47}$$

For a subwavelength structure, the plus sign in Eq. (S47) corresponds to the physical solution.

6. Normalization

According to Eq. (S38) and (S39), the expression for the pressure field for the dipole mode is

$$p_D = \begin{cases} A\sin\left(k_D r\theta\right) & \\ -A\sin\left(k_D r\left(\theta - \pi\right)\right) & \end{cases} \quad for \quad \begin{matrix} -\frac{\pi}{2} < \theta < \frac{\pi}{2} \\ \frac{\pi}{2} < \theta < \frac{3\pi}{2} \end{matrix} \tag{S48}$$

According to the definition of inner product in Eq. (S32), we have

$$\langle D | D \rangle = 2A^2\pi \tag{S49}$$

To normalize Eq. (S49), we set its value to be 1, then we have the amplitude of the dipole mode

$$A = \frac{1}{\sqrt{2\pi}} \tag{S50}$$

According to Eq. (S42), (S43) and (S45), the expression of the pressure field for the monopole mode is

$$p_M = \begin{cases} A_1 \cos\left(k_M r\theta\right) & -\frac{\pi}{2} < \theta < \frac{\pi}{2} \\ A_1 \frac{\cot\left(k_M r\pi/2\right)}{\cot\left(k_M r\pi/2\right) - Y_1'} \cos\left(k_M r(\theta - \pi)\right) + A_1 \frac{Y_1' \cot\left(k_M r\pi/2\right)}{\cot\left(k_M r\pi/2\right) - Y_1'} \sin\left(k_M r(\theta - \pi)\right) \quad for & \frac{\pi}{2} < \theta < \pi \\ A_1 \frac{\cot\left(k_M r\pi/2\right)}{\cot\left(k_M r\pi/2\right) - Y_1'} \cos\left(k_M r(\theta - \pi)\right) - A_1 \frac{Y_1' \cot\left(k_M r\pi/2\right)}{\cot\left(k_M r\pi/2\right) - Y_1'} \sin\left(k_M r(\theta - \pi)\right) & \pi < \theta < \frac{3\pi}{2} \end{cases}$$

(S51)

According the definition of inner product in Eq. (S32), and Eq. (S47), we have

$$\langle M | M \rangle = 2A_1^2 \pi \frac{1+Y_1'^2 + Y_1'^2 Y_2'^2 + Y_1' Y_2' \sqrt{1+Y_1'^2 + Y_1'^2 Y_2'^2}}{1+Y_1'^2} \tag{S52}$$

To normalize Eq. (S52), we set its value to be 1, then we have the amplitude of the monopole mode

$$A_1 = \sqrt{\frac{1+Y_1'^2}{2\pi\left(1+Y_1'^2 + Y_1'^2 Y_2'^2 + Y_1' Y_2' \sqrt{1+Y_1'^2 + Y_1'^2 Y_2'^2}\right)}} \,. \tag{S53}$$

7. Eigen-solution in the presence of bias

In the presence of bias flow, we assume the eigen-solution to be the linear superposition of monopole mode and dipole modes:

$$|\Psi\rangle = a_M |M\rangle + a_D |D\rangle \,. \tag{S54}$$

Here, a_M and a_D represent the complex amplitudes of monopole and dipole modal distributions, respectively, consistent with the main text. According to our previous results, we have

$$H_0 |M\rangle = \omega_M |M\rangle \tag{S55}$$

$$H_0 |D\rangle = \omega_D |D\rangle \,. \tag{S56}$$

By replacing Eqs. (S54), (S55) and (S56) into (S33), we get

$$a_M \delta H |M\rangle + a_D \delta H |D\rangle = (\omega - \omega_M) a_M |M\rangle + (\omega - \omega_D) a_D |D\rangle \,. \tag{S57}$$

Orthogonality requires

$$\langle D | M \rangle = \langle M | D \rangle = 0 \,. \tag{S58}$$

Applying the left ket $\langle M|$ and $\langle D|$ to Eq. (S57) and considering Eq. (S58), we find

$$\langle M|\delta H|M\rangle a_M + \langle M|\delta H|D\rangle a_D = (\omega - \omega_M) a_M \tag{S59}$$

$$\langle D|\delta H|M\rangle a_M + \langle D|\delta H|D\rangle a_D = (\omega - \omega_D) a_D . \tag{S60}$$

According to Eqs. (S34) and (S48) and (S51), we have

$$\langle M|\delta H|M\rangle = 0 \tag{S61}$$

$$\langle D|\delta H|D\rangle = 0 \tag{S62}$$

$$\langle D|\delta H|M\rangle = -\langle M|\delta H|D\rangle = iFU_0 \tag{S63}$$

Here F is a geometrical factor given by

$$F = -2A \frac{(B_1 - A_1) k_M \sin\left[\frac{\pi}{2}(k_M - k_D) r\right] + B_1 Y_1' \left(k_M \cos\left[\frac{\pi}{2} r (k_M - k_D)\right] - k_D \right)}{(k_M - k_D) r} . \tag{S64}$$

Specifically, when $k_M = k_D$, as in the case considered in the main text, we find

$$F = A(A_1 - B_1) k_M \pi - \frac{2AB_1 Y_1'}{r} . \tag{S65}$$

By replacing Eqs. (S61), (S62) and (S63) into Eqs. (S59) and (S60), we find

$$-iFU_0 a_D = (\omega - \omega_M) a_M \tag{S66}$$

$$iFU_0 a_M = (\omega - \omega_D) a_D \tag{S67}$$

Solving Eqs. (S66) and (S67), we can retrieve the eigen-frequency

$$\omega = \frac{(\omega_M + \omega_D) \pm \sqrt{(\omega_M - \omega_D)^2 + (2FU_0)^2}}{2} . \tag{S68}$$

Specifically, when $\omega_M = \omega_D$, we have

$$\omega = \omega_M \pm FU_0 , \tag{S69}$$

which confirms as expected that the modal degeneracy is lifted by the rotational flow, due to

Doppler shift, similar to[31]. From Eq. (S67) and (S68), we find the relation between a_M and a_D

$$a_M = \frac{(\omega_M - \omega_D) \pm \sqrt{(\omega_M - \omega_D)^2 + (2FU_0)^2}}{2iFU_0} a_D . \tag{S70}$$

Hence, we can rewrite Eq. (S54) as

$$|\Psi\rangle = \frac{(\omega_M - \omega_D) \pm \sqrt{(\omega_M - \omega_D)^2 + (2FU_0)^2}}{2iFU_0} a_D |M\rangle + a_D |D\rangle . \tag{S71}$$

Specifically, when $\omega_M = \omega_D$, we find

$$|\Psi\rangle = a_D \left(\pm i |M\rangle + |D\rangle \right) . \tag{S72}$$

8. Symmetry considerations

It is interesting to notice that our geometry consists of 3 cavities, with the third one breaking mirror symmetry along *y*. According to Eq. (S34), the operator δH applies $-i\Omega \frac{\partial}{\partial \theta}$ to the eigen-solutions for $|M\rangle$ and $|D\rangle$. Considering now Eq. (S61), we find

$$\langle M | \delta H | M \rangle = -i\Omega \langle M | \frac{\partial}{\partial \theta} | M \rangle = -i\Omega \int_{-\pi}^{\pi} \left[p_M^* \frac{dp_M}{d\theta} + \left(\rho_0 c_0 v_{\theta M}^* \right) \frac{d\left(\rho_0 c_0 v_{\theta M} \right)}{d\theta} \right] d\theta . \tag{S73}$$

We can separate Eq. (S73) into two parts, with the integral ranging from $-\pi \to 0$ and $0 \to \pi$, respectively:

$$\langle M | \delta H | M \rangle = -i\Omega \left[\int_{-\pi}^{0} \left[p_M^* \frac{dp_M}{d\theta} + \left(\rho_0 c_0 v_{\theta M}^* \right) \frac{d\left(\rho_0 c_0 v_{\theta M} \right)}{d\theta} \right] d\theta + \int_{0}^{\pi} \left[p_M^* \frac{dp_M}{d\theta} + \left(\rho_0 c_0 v_{\theta M}^* \right) \frac{d\left(\rho_0 c_0 v_{\theta M} \right)}{d\theta} \right] d\theta \right]$$

(S74)

For the monopole distribution, the pressure is symmetric, i.e., $p_M(\theta) = p_M(-\theta)$, but the first

derivative is anti-symmetric, i.e., $\frac{dp_M(\theta)}{d\theta} = -\frac{dp_M(-\theta)}{d\theta}$. Hence, if we combine them together, the first term is odd-symmetric, i.e., $p_M^*(\theta)\frac{dp_M(\theta)}{d\theta} = -p_M^*(-\theta)\frac{dp_M(-\theta)}{d\theta}$. For the same reason, we get $\rho_0 c_0 v_{\theta M}^*(\theta)\frac{d\left[\rho_0 c_0 v_{\theta M}(\theta)\right]}{d\theta} = -\rho_0 c_0 v_{\theta M}^*(-\theta)\frac{d\left[\rho_0 c_0 v_{\theta M}(-\theta)\right]}{d\theta}$. Therefore, the first term on the right side of Eq. (S74) is anti-symmetric with respect to the second term on the right side. Hence, we get $\langle M|\delta H|M\rangle = 0$, as shown in Eq. (S61). The basic physical reason is that $\langle M|$ and $\delta H|M\rangle$ are one even and the other one odd, resulting in a zero value of the integral. For the same reason, we also get $\langle D|\delta H|D\rangle = 0$. Next, we prove Eq. (S63), yielding $\langle D|\delta H|M\rangle = -\langle M|\delta H|D\rangle$, i.e., the origin of odd Willis coupling. We can write Eq. (S63) as

$$\langle D|\delta H|M\rangle = -i\Omega\langle D|\frac{\partial}{\partial\theta}|M\rangle = -i\Omega\int_{-\pi}^{\pi}\left[p_D^*\frac{dp_M}{d\theta} + \left(\rho_0 c_0 v_{\theta D}^*\right)\frac{d\left(\rho_0 c_0 v_{\theta M}\right)}{d\theta}\right]d\theta . \quad \text{(S75)}$$

We get

$$\langle D|\delta H|M\rangle = -i\Omega\left[\int_{-\pi}^{\pi} p_D^* dp_M + \int_{-\pi}^{\pi}\left(\rho_0 c_0 v_{\theta D}^*\right)d\left(\rho_0 c_0 v_{\theta M}\right)\right] \quad \text{(S76)}$$

and

$$\langle D|\delta H|M\rangle = -i\Omega\left[p_D^* p_M\Big|_{-\pi}^{\pi} - \int_{-\pi}^{\pi} p_M dp_D^* + \left(\rho_0 c_0 v_{\theta D}^*\right)\left(\rho_0 c_0 v_{\theta M}\right)\Big|_{-\pi}^{\pi} - \int_{-\pi}^{\pi}\left(\rho_0 c_0 v_{\theta M}\right)d\left(\rho_0 c_0 v_{\theta D}^*\right)\right]$$

(S77)

Since the first and third terms on the right side of (S77) are zero, we have

$$\langle D|\delta H|M\rangle = i\Omega\int_{-\pi}^{\pi}\left[p_M\frac{dp_D^*}{d\theta} + \left(\rho_0 c_0 v_{\theta M}\right)\frac{d\left(\rho_0 c_0 v_{\theta D}^*\right)}{d\theta}\right]d\theta \quad \text{(S78)}$$

According to Eq. (S50) and (S53), since p_M and p_D are purely real, $p_M\frac{dp_D^*}{d\theta} = p_M^*\frac{dp_D}{d\theta}$. For the

same reason, we have $\left(\rho_0 c_0 v_{\theta M}\right)\dfrac{d\left(\rho_0 c_0 v_{\theta D}^*\right)}{d\theta}=\left(\rho_0 c_0 v_{\theta M}^*\right)\dfrac{d\left(\rho_0 c_0 v_{\theta D}\right)}{d\theta}$.

Hence Eq. (S78) can be rewritten as

$$\langle D|\delta H|M\rangle = i\Omega\int_{-\pi}^{\pi}\left[p_M^*\frac{dp_D}{d\theta}+\left(\rho_0 c_0 v_{\theta M}^*\right)\frac{d\left(\rho_0 c_0 v_{\theta D}\right)}{d\theta}\right]d\theta = -\langle M|\delta H|D\rangle \tag{S79}$$

In general, $\langle D|\delta H|M\rangle \neq 0$ because $\langle D|$ and $\delta H|M\rangle$ are both even or both odd, as shown schematically in Fig. S2 a,b,c.

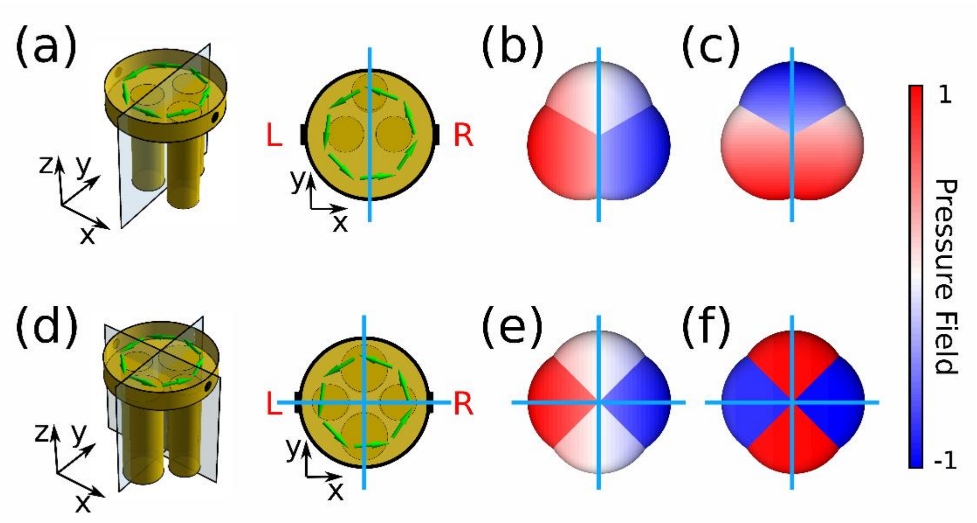

Fig. S2. a. A structure with 3 cylinders which only preserve mirror symmetry in *x*-direction. **b,c.** Eigen modes of dipole and monopole. **d.** A structure with 4 cylinders which preserve mirror symmetries in both *x*- and *y*-directions. **e,f.** Eigen modes of dipole and monopole.

However, if we consider a scatterer with four cylinders, as in Fig. S2 d,e,f, with both *x*- and *y*-mirror symmetry, $\langle D|\delta H|M\rangle$ becomes zero. For example, as shown in figure S2 d,e,f, in the *x*-direction, the dipole mode is odd, but the monopole mode is even. And hence $\langle D|$ and $\frac{\partial}{\partial\theta}|M\rangle$ are both odd or both even. However, if we look in the *y*-direction, both dipole and monopole modes

are even, resulting in $\langle D|$ and $\frac{\partial}{\partial\theta}|M\rangle$ to be one odd and the other one even. And hence $\langle D|\delta H|M\rangle = 0$. We conclude that purely odd Willis coupling can be achieved only when the geometry is mirror symmetric in *x*, but with broken symmetry in *y*.

9. S-matrix and polarizability tensor

In this section, we use again the SI units, and connect the polarizability tensor with the scattering matrix of the system. The modified unit system defined in the beginning of the supplementary materials and used in the main paper will be indicated with a prime. For example, M indicates the monopole in SI unit, and M' indicates the monopole in modified units.

For a monopole source located at the origin, the wave equation can be expressed as

$$\nabla^2 p + k^2 p = \omega^2 M \delta(\vec{r}). \tag{S80}$$

A common solution of Eq. (S80) is

$$p_{M-} = -i\frac{\omega^2 M}{4} H_0^{(1)}(k_0 r), \tag{S81}$$

which corresponds to an outgoing monopole wave. The corresponding incoming monopole wave, also solution of (S80) is given by

$$p_{M+} = i\frac{\omega^2 M}{4} H_0^{(2)}(k_0 r). \tag{S82}$$

For an acoustic scatterer placed in a pressure field, the incident pressure is a standing wave, superposition of an outgoing and incoming monopole waves:

$$p_{inc} = p_{loc} J_0(k_0 r) = \frac{p_{loc}}{2}\left[H_0^{(1)}(k_0 r) + H_0^{(2)}(k_0 r)\right]. \tag{S83}$$

We can rewrite Eq. (S83) as

$$p_{inc} = i\frac{\omega^2 M_+}{2} J_0(k_0 r) = i\frac{\omega^2 M_+}{4} H_0^{(1)}(k_0 r) + i\frac{\omega^2 M_+}{4} H_0^{(2)}(k_0 r)\,. \quad \text{(S84)}$$

Here the local pressure on the scatterer is:

$$p_{loc} = i\frac{\omega^2 M_+}{2}\,. \quad \text{(S85)}$$

From Eq. (S85), we find that an incident pressure wave with amplitude p_{loc} can be regarded as the superposition of an incoming monopole wave with amplitude M_+ and an outgoing monopole wave with amplitude $-M_+$. For a non-bianisotropic scatterer placed in such a pressure standing wave, the total field can be expressed as

$$p_{tot} = p_{loc} J_0(k_0 r) - i\frac{\omega^2 M}{4} H_0^{(1)}(k_0 r)\,. \quad \text{(S86)}$$

The first term indicates that the incident field, and the second term indicates the scattered field, which is an outgoing monopole wave with amplitude M. By considering Eq. (S85), we can write Eq. (S86) as

$$p_{tot} = i\frac{\omega^2 M_+}{4}\left[H_0^{(1)}(k_0 r) + H_0^{(2)}(k_0 r)\right] - i\frac{\omega^2 M}{4} H_0^{(1)}(k_0 r)\,. \quad \text{(S87)}$$

By rearranging Eq. (S87), we can rewrite it as

$$p_t = -i\frac{\omega^2 M_-}{4} H_0^{(1)}(k_0 r) + i\frac{\omega^2 M_+}{4} H_0^{(2)}(k_0 r)\,. \quad \text{(S88)}$$

Here we set

$$M_- = M - M_+\,. \quad \text{(S89)}$$

The total field expressed in Eq. (S88) can be regarded as the superposition of an outgoing monopole with amplitude M_- and an incoming monopole with amplitude M_+. Although Eqs. (S86) and (S88) represent the same field, the physical picture is quite different. In the traditional

scattering problem expressed by Eq. (S86), we relate the scattered monopole and dipole as a function of incident pressure and velocity, using modified units, we have

$$\begin{pmatrix} M' \\ \vec{D}' \end{pmatrix} = \boldsymbol{\alpha}' \begin{pmatrix} p'_{loc} \\ \vec{v}'_{loc} \end{pmatrix} = \begin{pmatrix} \alpha^{pp\prime} & \boldsymbol{\alpha}^{pv\prime} \\ \boldsymbol{\alpha}^{vp\prime} & \boldsymbol{\alpha}^{vv\prime} \end{pmatrix} \begin{pmatrix} p'_{loc} \\ \vec{v}'_{loc} \end{pmatrix} \tag{S90}$$

From Eq. (S88), we can also relate the divergence monopole and dipole as a function of the incoming wave:

$$\begin{pmatrix} M'_- \\ \vec{D}'_- \end{pmatrix} = \mathbf{s}' \begin{pmatrix} M'_+ \\ \vec{D}'_+ \end{pmatrix} = \begin{pmatrix} s^{pp\prime} & \mathbf{s}^{pv\prime} \\ \mathbf{s}^{vp\prime} & \mathbf{s}^{vv\prime} \end{pmatrix} \begin{pmatrix} M'_+ \\ \vec{D}'_+ \end{pmatrix}. \tag{S91}$$

From Eq. (S89), we know that $M_-{}' = M' - M_+{}'$.

Applying the same procedure, we can find a similar relation for the dipolar scattering, hence we have

$$\begin{pmatrix} M' \\ \vec{D}' \end{pmatrix} = \begin{pmatrix} M'_+ \\ \vec{D}'_+ \end{pmatrix} + \begin{pmatrix} M'_- \\ \vec{D}'_- \end{pmatrix} \tag{S92}$$

We can write Eq. (S85) as

$$p'_{loc} = \frac{p_{loc}}{\sqrt{2}} = -i\,\frac{\omega^2\left(-\sqrt{2}M_+\right)}{4} = -i\,\frac{\omega^2 M'_+}{4}. \tag{S93}$$

Applying the same procedure, we find that this relation applies to a velocity standing wave excitation and dipole waves, hence we have

$$\begin{pmatrix} p'_{loc} \\ \vec{v}'_{loc} \end{pmatrix} = -i\,\frac{\omega^2}{4} \begin{pmatrix} M'_+ \\ \vec{D}'_+ \end{pmatrix}. \tag{S94}$$

By replacing Eqs. (S92) and (S94) into Eq. (S90), we get

$$\begin{pmatrix} M'_- \\ \vec{D}'_- \end{pmatrix} = \left(-\mathbf{I} - i\,\frac{\omega^2}{4}\boldsymbol{\alpha} \right) \begin{pmatrix} M'_+ \\ \vec{D}'_+ \end{pmatrix}. \tag{S95}$$

Here $\mathbf{I}$ is the unit matrix. By comparing Eq. (S95) with Eq. (S91), we find the relation between the polarization tensor $\boldsymbol{\alpha}$ and the scattering matrix:

$$\mathbf{s}' = -\mathbf{I} - i\frac{\omega^2}{4}\boldsymbol{\alpha}' . \tag{S96}$$

Specifically, when $\boldsymbol{\alpha} = 0$, i.e., in the absence of the scatterer, the S-Matrix satisfies the expected relation

$$\mathbf{s}' = -\mathbf{I} \tag{S97}$$

10. Scattering analysis

In order to model the scattering of our device in terms of its polarizability tensor, we develop a temporal coupled-mode theory for the cavity modes excited by monopole and dipole scattering harmonics. We generally assume that the scatterer supports a monopolar and a dipolar mode, with complex amplitude a_M and a_D, which can be excited by incoming monopole and dipole harmonics respectively, and which decay into monopolar and dipolar outgoing harmonics. The equations of motion can be then written as

$$\frac{d}{dt}\begin{pmatrix} a_M \\ a_D \end{pmatrix} = \begin{pmatrix} -i\omega_M - \gamma_M & -FU_0 \\ FU_0 & -i\omega_D - \gamma_D \end{pmatrix}\begin{pmatrix} a_M \\ a_D \end{pmatrix} + \begin{pmatrix} \sqrt{2\gamma_M} & 0 \\ 0 & i\sqrt{2\gamma_D} \end{pmatrix}\begin{pmatrix} S_{+M} \\ S_{+D} \end{pmatrix} \tag{S98}$$

$$\begin{pmatrix} S_{-M} \\ S_{-D} \end{pmatrix} = \begin{pmatrix} -1 & 0 \\ 0 & -1 \end{pmatrix}\begin{pmatrix} S_{+M} \\ S_{+D} \end{pmatrix} + \begin{pmatrix} \sqrt{2\gamma_M} & 0 \\ 0 & -i\sqrt{2\gamma_D} \end{pmatrix}\begin{pmatrix} a_M \\ a_D \end{pmatrix}, \tag{S99}$$

where S_{+M}, S_{-M} are the incoming and outgoing monopole harmonics, and S_{+D}, S_{-D} are the corresponding dipole harmonics. Here ω_M and ω_D represent the angular frequencies of the monopole and dipole mode, γ_M and γ_D represent their decay rates, U_0 is the rotational flow velocity, and F is a geometrical factor.

From Eq. (S98), we get

$$\begin{pmatrix} a_M \\ a_D \end{pmatrix} = \begin{pmatrix} i\left(\omega_M-\omega\right)+\gamma_M & FU_0 \\ -FU_0 & i\left(\omega_D-\omega\right)+\gamma_D \end{pmatrix}^{-1} \begin{pmatrix} \sqrt{2\gamma_M} & 0 \\ 0 & i\sqrt{2\gamma_D} \end{pmatrix} \begin{pmatrix} S_{+M} \\ S_{+D} \end{pmatrix}. \tag{S100}$$

By replacing Eq. (S100) into Eq. (S99), we get

$$\begin{pmatrix} S_{-M} \\ S_{-D} \end{pmatrix} = \left[-\overleftrightarrow{\mathbf{I}} + \begin{pmatrix} \sqrt{2\gamma_M} & 0 \\ 0 & -i\sqrt{2\gamma_D} \end{pmatrix} \begin{pmatrix} i\left(\omega_M-\omega\right)+\gamma_M & FU_0 \\ -FU_0 & i\left(\omega_D-\omega\right)+\gamma_D \end{pmatrix}^{-1} \begin{pmatrix} \sqrt{2\gamma_M} & 0 \\ 0 & i\sqrt{2\gamma_D} \end{pmatrix} \right] \begin{pmatrix} S_{+M} \\ S_{+D} \end{pmatrix} \tag{S101}$$

After simplification, we can rewrite Eq. (S101) as

$$\begin{pmatrix} S_{-M} \\ S_{-D} \end{pmatrix} = \left[-\overleftrightarrow{\mathbf{I}} + \begin{pmatrix} \frac{2\gamma_M\left[\gamma_D - i(\omega-\omega_D)\right]}{\left(FU_0\right)^2 - \left(\omega-\omega_D+i\gamma_D\right)\left(\omega-\omega_M+i\gamma_M\right)} & \frac{-i2FU_0\sqrt{\gamma_M\gamma_D}}{\left(FU\right)_0^2 - \left(\omega-\omega_D+i\gamma_D\right)\left(\omega-\omega_M+i\gamma_M\right)} \\ \frac{-i2FU_0\sqrt{\gamma_M\gamma_D}}{\left(FU_0\right)^2 - \left(\omega-\omega_D+i\gamma_D\right)\left(\omega-\omega_M+i\gamma_M\right)} & \frac{2\gamma_d\left[\gamma_M - i(\omega-\omega_M)\right]}{\left(FU_0\right)^2 - \left(\omega-\omega_D+i\gamma_D\right)\left(\omega-\omega_M+i\gamma_M\right)} \end{pmatrix} \right] \begin{pmatrix} S_{+M} \\ S_{+D} \end{pmatrix} \tag{S102}$$

By comparing Eq. (S95) with (S102), we find

$$\boldsymbol{\alpha}' = i\frac{4}{\omega^2} \begin{pmatrix} \frac{2\gamma_M\left[\gamma_D - i(\omega-\omega_D)\right]}{\left(FU_0\right)^2 - \left(\omega-\omega_D+i\gamma_D\right)\left(\omega-\omega_M+i\gamma_M\right)} & \frac{-i2FU_0\sqrt{\gamma_M\gamma_D}}{\left(FU\right)_0^2 - \left(\omega-\omega_D+i\gamma_D\right)\left(\omega-\omega_M+i\gamma_M\right)} \\ \frac{-i2FU_0\sqrt{\gamma_M\gamma_D}}{\left(FU_0\right)^2 - \left(\omega-\omega_D+i\gamma_D\right)\left(\omega-\omega_M+i\gamma_M\right)} & \frac{2\gamma_d\left[\gamma_M - i(\omega-\omega_M)\right]}{\left(FU_0\right)^2 - \left(\omega-\omega_D+i\gamma_D\right)\left(\omega-\omega_M+i\gamma_M\right)} \end{pmatrix} \tag{S103}$$

And hence

$$\alpha_x^{pv\prime} = \alpha_x^{vp\prime} = \frac{8}{\omega^2}\frac{FU_0\sqrt{\gamma_M\gamma_D}}{\left(FU_0\right)^2 - \left(\omega-\omega_M+i\gamma_M\right)\left(\omega-\omega_D+i\gamma_D\right)}\ . \tag{S104}$$

Specifically, when $\omega = \omega_M = \omega_D$,

$$\alpha_x^{pv\prime} = \alpha_x^{vp\prime} = \frac{8}{\omega^2}\frac{FU_0\sqrt{\gamma_M\gamma_D}}{\left(FU_0\right)^2+\gamma_M\gamma_D} \quad . \tag{S105}$$

We can also simplify Eq. (S100) as

$$\begin{pmatrix} a_M \\ a_D \end{pmatrix} = \begin{pmatrix} \dfrac{-i\sqrt{2\gamma_M}\left[\omega-\omega_D+i\gamma_D\right]}{\left(FU_0\right)^2-\left(\omega-\omega_D+i\gamma_D\right)\left(\omega-\omega_M+i\gamma_M\right)} & \dfrac{-iFU_0\sqrt{2\gamma_D}}{\left(FU\right)_0^{\;2}-\left(\omega-\omega_D+i\gamma_D\right)\left(\omega-\omega_M+i\gamma_M\right)} \\ \dfrac{FU_0\sqrt{2\gamma_M}}{\left(FU_0\right)^2-\left(\omega-\omega_D+i\gamma_D\right)\left(\omega-\omega_M+i\gamma_M\right)} & \dfrac{\sqrt{2\gamma_D}\left[\omega-\omega_M+i\gamma_M\right]}{\left(FU_0\right)^2-\left(\omega-\omega_D+i\gamma_D\right)\left(\omega-\omega_M+i\gamma_M\right)} \end{pmatrix} \begin{pmatrix} S_{+M} \\ S_{+D} \end{pmatrix} \tag{S106}$$

Specifically, when $\omega = \omega_M = \omega_D$,

$$\begin{pmatrix} a_M \\ a_D \end{pmatrix} = \begin{pmatrix} \dfrac{\sqrt{2\gamma_M\gamma_D^2}}{\left(FU_0\right)^2+\gamma_D\gamma_M} & \dfrac{-iFU_0\sqrt{2\gamma_D}}{\left(FU_0\right)^2+\gamma_D\gamma_M} \\ \dfrac{FU_0\sqrt{2\gamma_M}}{\left(FU_0\right)^2+\gamma_D\gamma_M} & \dfrac{i\sqrt{2\gamma_M^2\gamma_D}}{\left(FU_0\right)^2+\gamma_D\gamma_M} \end{pmatrix} \begin{pmatrix} S_{+M} \\ S_{+D} \end{pmatrix} \tag{S107}$$

and according to Eq. (S94)

$$\begin{pmatrix} a_M \\ a_D \end{pmatrix} = \frac{4}{\omega^2} \begin{pmatrix} \dfrac{i\sqrt{2\gamma_M\gamma_D^2}}{\left(FU_0\right)^2+\gamma_D\gamma_M} & \dfrac{FU_0\sqrt{2\gamma_D}}{\left(FU_0\right)^2+\gamma_D\gamma_M} \\ \dfrac{iFU_0\sqrt{2\gamma_M}}{\left(FU_0\right)^2+\gamma_D\gamma_M} & \dfrac{-\sqrt{2\gamma_M^2\gamma_D}}{\left(FU_0\right)^2+\gamma_D\gamma_M} \end{pmatrix} \begin{pmatrix} p'_{loc} \\ \vec{v}'_{loc} \end{pmatrix}. \tag{S108}$$

Specifically, at the optimal rotational velocity, i.e. when $U_0 = \sqrt{\gamma_M\gamma_D}\big/F$

$$\begin{pmatrix} a_M \\ a_D \end{pmatrix} = \frac{4}{\omega^2} \begin{pmatrix} \dfrac{i}{\sqrt{2\gamma_M}} & \dfrac{1}{\sqrt{2\gamma_M}} \\ \dfrac{i}{\sqrt{2\gamma_D}} & \dfrac{-1}{\sqrt{2\gamma_D}} \end{pmatrix} \begin{pmatrix} p'_{loc} \\ \vec{v}'_{loc} \end{pmatrix} \quad . \tag{S109}$$

11. Role of asymmetries and loss

We can now introduce an asymmetry to the left and right cavities. In general, we can modify the sound speed of the left cavity as $c_0(1+\beta_L)$, the middle cavity as $c_0(1+\beta_M)$, and of the right cavity as $c_0(1+\beta_R)$. Here β can either be real and imaginary, even complex. When β is imaginary, it indicates that the system is non-Hermitian. According to Eq. (S37)

$$Y_n+\delta Y_n=-i\frac{\pi r_h^2}{r\Delta r\rho_0 c_0}\tan\left(\frac{\omega}{c_0(1+\beta)}l_n\right). \tag{S110}$$

When $\beta\ll 1$ is satisfied

$$Y_n+\delta Y_n=-i\frac{\pi r_h^2}{r\Delta r\rho_0 c_0}\tan\left(kl_n\right)+i\beta\frac{kl_n\pi r_h^2\left[1+\tan^2\left(kl_n\right)\right]}{r\Delta r\rho_0 c_0}. \tag{S111}$$

Hence,

$$\delta Y_{n(L,M,R)}=i\beta_{(L,M,R)}\frac{kl_n\pi r_h^2\left[1+\tan^2\left(kl_n\right)\right]}{r\Delta r\rho_0 c_0}. \tag{S112}$$

The impedance equation in (S26) is changed to

$$Y=\left(Y_1+\delta Y_{1M}\right)\delta\left(\theta-\pi\right)+\left(Y_2+\delta Y_{2L}\right)\delta\left(\theta+\frac{\pi}{2}\right)+\left(Y_2+\delta Y_{2R}\right)\delta\left(\theta-\frac{\pi}{2}\right). \tag{S113}$$

Here we keep H_0 as Eq. (S31) as the constant part, and calculate δH in Eq. (S34) as

$$\delta H=\begin{pmatrix}-i\frac{\delta Y\rho_0 c_0^2}{h_w}-i\Omega\frac{\partial}{\partial\theta} & 0\\ 0 & -i\Omega\frac{\partial}{\partial\theta}\end{pmatrix}. \tag{S114}$$

By replacing the new expression of δH into Eq. (S61), (S62) and (S63), we get

$$\left\langle M\left|\delta H\right|M\right\rangle=-i\left(\delta Y_{2L}+\delta Y_{2R}\right)\frac{\rho_0 c_0^2}{h_w}\left[A_1\cos\left(k_M r\frac{\pi}{2}\right)\right]^2-i\delta Y_{1M}\frac{\rho_0 c_0^2}{h_w}B_1^2 \tag{S115}$$

$$\langle D|\delta H|D\rangle = -i\left(\delta Y_{2L}+\delta Y_{2R}\right)\frac{\rho_0 c_0^2}{h_w}\left[A\sin\left(k_D r\frac{\pi}{2}\right)\right]^2 \tag{S116}$$

$$\langle D|\delta H|M\rangle = iFU_0 + i\frac{\left(\delta Y_{2L}-\delta Y_{2R}\right)\rho_0 c_0^2}{h_w}AA_1\sin\left(k_D r\frac{\pi}{2}\right)\cos\left(k_D r\frac{\pi}{2}\right) \tag{S117}$$

$$\langle M|\delta H|D\rangle = -iFU_0 + i\frac{\left(\delta Y_{2L}-\delta Y_{2R}\right)\rho_0 c_0^2}{h_w}AA_1\sin\left(k_D r\frac{\pi}{2}\right)\cos\left(k_D r\frac{\pi}{2}\right) \tag{S118}$$

Since $A_1\cos\left(k_M r\frac{\pi}{2}\right)$ and $A\sin\left(k_M r\frac{\pi}{2}\right)$ represent the amplitude at the outlets for monopole and dipole, respectively, their relation to the decay rate are

$$\frac{A_1\cos\left(k_M r\frac{\pi}{2}\right)}{A\sin\left(k_D r\frac{\pi}{2}\right)} = \sqrt{\frac{\gamma_M}{\gamma_D}} \tag{S119}$$

And hence, we can write Eq. (S115), (S116), (S117), (S118) according to Eq. (S50) and (S112)

$$\langle M|\delta H|M\rangle = \left(\beta_L+\beta_R\right)X + \beta_M X' \tag{S120}$$

$$\langle D|\delta H|D\rangle = \left(\beta_L+\beta_R\right)\frac{\gamma_D}{\gamma_M}X \tag{S121}$$

$$\langle D|\delta H|M\rangle = iFU_0 - \left(\beta_L-\beta_R\right)\sqrt{\frac{\gamma_D}{\gamma_M}}X \tag{S122}$$

$$\langle M|\delta H|D\rangle = -iFU_0 - \left(\beta_L-\beta_R\right)\sqrt{\frac{\gamma_D}{\gamma_M}}X\ . \tag{S123}$$

Here

$$X = \frac{kl_2 r_h^2 c_0\left[1+\tan^2\left(kl_2\right)\right]}{h_w r\Delta r}\frac{\gamma_M}{\gamma_D}\sin^2\left(k_D r\frac{\pi}{2}\right) \tag{S124}$$

$$X' = \frac{kl_n \pi r_h^2 c_0\left[1+\tan^2\left(kl_n\right)\right]}{h_w r\Delta r}B_1^2 \tag{S125}$$

X is a purely real and positive number. According to Eq. (S59) and (S60), the coupled mode equation including the presence of asymmetry, or loss, can be written as

$$\frac{d}{dt}\begin{pmatrix} a_M \\ a_D \end{pmatrix} = \begin{pmatrix} -i\omega_M - \gamma_M - i\left(\beta_L + \beta_R\right)X - i\beta_M X' & -FU_0 + i\left(\beta_L - \beta_R\right)\sqrt{\frac{\gamma_D}{\gamma_M}}X \\ FU_0 + i\left(\beta_L - \beta_R\right)\sqrt{\frac{\gamma_D}{\gamma_M}}X & -i\omega_D - \gamma_D - i\left(\beta_L + \beta_R\right)\frac{\gamma_D}{\gamma_M}X \end{pmatrix}\begin{pmatrix} a_M \\ a_D \end{pmatrix} + \begin{pmatrix} \sqrt{2\gamma_M} & 0 \\ 0 & i\sqrt{2\gamma_D} \end{pmatrix}\begin{pmatrix} S_{+M} \\ S_{+D} \end{pmatrix} \tag{S126}$$

$$\begin{pmatrix} S_{-M} \\ S_{-D} \end{pmatrix} = \begin{pmatrix} -1 & 0 \\ 0 & -1 \end{pmatrix}\begin{pmatrix} S_{+M} \\ S_{+D} \end{pmatrix} + \begin{pmatrix} \sqrt{2\gamma_M} & 0 \\ 0 & -i\sqrt{2\gamma_D} \end{pmatrix}\begin{pmatrix} a_M \\ a_D \end{pmatrix} \tag{S127}$$

$$\boldsymbol{\alpha}' = i\frac{4}{\omega^2}\begin{pmatrix} \sqrt{2\gamma_M} & 0 \\ 0 & -i\sqrt{2\gamma_D} \end{pmatrix}\begin{pmatrix} i\left(\omega_M - \omega\right) + \gamma_M + i\left(\beta_L + \beta_R\right)X + i\beta_M X' & FU_0 - i\left(\beta_L - \beta_R\right)\sqrt{\frac{\gamma_D}{\gamma_M}}X \\ -FU_0 - i\left(\beta_L - \beta_R\right)\sqrt{\frac{\gamma_D}{\gamma_M}}X & i\left(\omega_D - \omega\right) + \gamma_D + i\left(\beta_L + \beta_R\right)\frac{\gamma_D}{\gamma_M}X \end{pmatrix}^{-1}\begin{pmatrix} \sqrt{2\gamma_M} & 0 \\ 0 & i\sqrt{2\gamma_D} \end{pmatrix} \tag{S128}$$

Specifically, when $\omega = \omega_M = \omega_D$:

$$\boldsymbol{\alpha}' = \frac{8}{\omega^2}\begin{pmatrix} \frac{i\gamma_M\gamma_D - \left(\beta_L + \beta_R\right)X\gamma_D}{\left(FU_0\right)^2 + \frac{\gamma_D}{\gamma_M}\left[\left(\gamma_M + 2i\beta_L X\right)\left(\gamma_M + 2i\beta_R X\right) + i\beta_M\left(\gamma_M + i\left(\beta_L + \beta_R\right)X\right)X'\right]} & \frac{FU_0\sqrt{\gamma_M\gamma_D} - i\left(\beta_L - \beta_R\right)\gamma_D X}{\left(FU_0\right)^2 + \frac{\gamma_D}{\gamma_M}\left[\left(\gamma_M + 2i\beta_L X\right)\left(\gamma_M + 2i\beta_R X\right) + i\beta_M\left(\gamma_M + i\left(\beta_L + \beta_R\right)X\right)X'\right]} \\ \frac{FU_0\sqrt{\gamma_M\gamma_D} + i\left(\beta_L - \beta_R\right)\gamma_D X}{\left(FU_0\right)^2 + \frac{\gamma_D}{\gamma_M}\left[\left(\gamma_M + 2i\beta_L X\right)\left(\gamma_M + 2i\beta_R X\right) + i\beta_M\left(\gamma_M + i\left(\beta_L + \beta_R\right)X\right)X'\right]} & \frac{i\gamma_M\gamma_D - \left(\beta_L + \beta_R\right)X\gamma_D - \beta_M X'\gamma_D}{\left(FU_0\right)^2 + \frac{\gamma_D}{\gamma_M}\left[\left(\gamma_M + 2i\beta_L X\right)\left(\gamma_M + 2i\beta_R X\right) + i\beta_M\left(\gamma_M + i\left(\beta_L + \beta_R\right)X\right)X'\right]} \end{pmatrix} \tag{S129}$$

In the main paper, we consider $\beta_R = \beta_M = 0$, and hence

$$\boldsymbol{\alpha}' = \frac{8}{\omega^2}\begin{pmatrix} \frac{i\gamma_M\gamma_D - \beta_L X\gamma_D}{\left(FU_0\right)^2 + \gamma_D\left(\gamma_M + 2i\beta_L X\right)} & \frac{FU_0\sqrt{\gamma_M\gamma_D} - i\beta_L X\gamma_D}{\left(FU_0\right)^2 + \gamma_D\left(\gamma_M + 2i\beta_L X\right)} \\ \frac{FU_0\sqrt{\gamma_M\gamma_D} + i\beta_L X\gamma_D}{\left(FU_0\right)^2 + \gamma_D\left(\gamma_M + 2i\beta_L X\right)} & \frac{i\gamma_M\gamma_D - \beta_L X\gamma_D}{\left(FU_0\right)^2 + \gamma_D\left(\gamma_M + 2i\beta_L X\right)} \end{pmatrix} \tag{S130}$$

at the optimal flow speed $U_0 = \sqrt{\gamma_M\gamma_D}\big/F$

$$\boldsymbol{\alpha}' = \frac{4}{\omega^2}\begin{pmatrix} i & \frac{\gamma_M - i\beta_L X}{\gamma_M + i\beta_L X} \\ 1 & i \end{pmatrix}. \tag{S131}$$

12. Location of the pressure node in Fig. 1e

If the inclusion is placed in a pressure field, i.e. $\begin{pmatrix} p'_{loc} & \vec{v}'_{loc} \end{pmatrix}^T = \begin{pmatrix} 1 & 0 \end{pmatrix}$, according to Eq. (S109), the modal distribution inside the cavity is

$$\begin{pmatrix} a_M \\ a_D \end{pmatrix} = \frac{4}{\omega^2}\begin{pmatrix} \frac{i}{\sqrt{2\gamma_M}} & \frac{1}{\sqrt{2\gamma_M}} \\ \frac{i}{\sqrt{2\gamma_D}} & \frac{-1}{\sqrt{2\gamma_D}} \end{pmatrix}\begin{pmatrix} 1 \\ 0 \end{pmatrix} = \frac{4}{\omega^2}\begin{pmatrix} \frac{i}{\sqrt{2\gamma_M}} \\ \frac{i}{\sqrt{2\gamma_D}} \end{pmatrix}. \tag{S132}$$

According to Eqs. (S38) and (S42), the pressure distribution between $-\frac{\pi}{2} < \theta < \frac{\pi}{2}$ can be expressed as

$$p_{cavity} = a_M A_1 \cos(k_M r\theta) + a_D A \sin(k_D r\theta) \tag{S133}$$

At $\theta = -\frac{\pi}{2}$, the pressure is

$$p_{cavity}\left(-\frac{\pi}{2}\right) = a_M A_1 \cos\left(k_M r\frac{\pi}{2}\right) - a_D A \sin\left(k_D r\frac{\pi}{2}\right) \tag{S134}$$

According to Eq. (S119), we can set

$$A_1 \cos\left(k_M r\frac{\pi}{2}\right) = \xi\sqrt{\gamma_M} \tag{S135}$$

$$A \sin\left(k_D r\frac{\pi}{2}\right) = \xi\sqrt{\gamma_D} \tag{S136}$$

with ξ being arbitrary proportionality coefficient. Replace Eqs. (S132), (S135) and (S136) into Eq. (S134), we have

$$p_{cavity}\left(-\frac{\pi}{2}\right) = \frac{4}{\omega^2}\frac{i}{\sqrt{2\gamma_M}}\xi\sqrt{\gamma_M} - \frac{4}{\omega^2}\frac{i}{\sqrt{2\gamma_D}}\xi\sqrt{\gamma_D} = 0 \tag{S137}$$

If the inclusion is placed in a velocity field, i.e., $\begin{pmatrix} p'_{loc} & \vec{v}'_{loc} \end{pmatrix}^T = \begin{pmatrix} 0 & 1 \end{pmatrix}$, according to Eq. (S109), the mode distribution inside the cavity is

$$\begin{pmatrix} a_M \\ a_D \end{pmatrix} = \frac{4}{\omega^2} \begin{pmatrix} \frac{i}{\sqrt{2\gamma_M}} & \frac{1}{\sqrt{2\gamma_M}} \\ \frac{i}{\sqrt{2\gamma_D}} & \frac{-1}{\sqrt{2\gamma_D}} \end{pmatrix} \begin{pmatrix} 0 \\ 1 \end{pmatrix} = \frac{4}{\omega^2} \begin{pmatrix} \frac{1}{\sqrt{2\gamma_M}} \\ \frac{-1}{\sqrt{2\gamma_D}} \end{pmatrix} \tag{S138}$$

At the opposite side, $\theta = \frac{\pi}{2}$, the pressure is

$$p_{cavity}\left(\frac{\pi}{2}\right) = a_M A_1 \cos\left(k_M r \frac{\pi}{2}\right) + a_D A \sin\left(k_D r \frac{\pi}{2}\right) \tag{S139}$$

Replacing Eqs. (S132), (S135) and (S136) into Eq. (S139), we have

$$p_{cavity}\left(\frac{\pi}{2}\right) = \frac{4}{\omega^2} \frac{1}{\sqrt{2\gamma_M}} \xi \sqrt{\gamma_M} + \frac{4}{\omega^2} \frac{-1}{\sqrt{2\gamma_D}} \xi \sqrt{\gamma_D} = 0 \tag{S140}$$

13. Duality in scattering

Here we validate with full-wave simulations the experimental results shown in Fig. 4 of the main text and further discuss the duality between even and odd Willis scatterers. We confirm the exotic response of purely odd Willis scatterers, and compare them with the case of a symmetric scatterer with no Willis coupling. In the left column, we analyze the total scattering width (Fig. S3b) and scattering pattern at resonance (Fig. S3c) of a symmetric scatterer for oppositely propagating excitations. As expected, the response is totally symmetric. In the middle column, we analyze an even Willis scatterer. While the scattering pattern at resonance (Fig. S3f) can be drastically different, the forward scattering is necessarily identical because of reciprocity. The optical theorem then ensures that the total scattered power and scattering width (Fig. S3e) for opposite excitations is necessarily identical at all frequencies. This result applies to any reciprocal scatterer. While the backward scattering can be widely different, the forward scattering and total scattering width must

be identical for opposite excitations. The right column analyzes the dual scenario of an odd Willis scatterer. Here, the backward scattering is identical, but forward scattering can be widely different (Fig. S3i), implying that at resonance the total scattering width for opposite excitations (Fig. S3h) can be remarkably different.

14. Scattering pattern and total scattering width

For the subwavelength scatterers, the scattered fields can be expressed as:

$$p = p_M + p_{Dx} + p_{Dy} = -\frac{ik_0^2 c_0^2}{4} \cdot M \cdot H_0^{(1)}\left(k_0 r\right) - \frac{ik_0^3 c_0^2}{4} \cdot H_1^{(1)}\left(k_0 r\right) \cdot \left(D_x \cos\theta + D_y \sin\theta\right) \tag{S141}$$

By taking the asymptotic form of Hankel functions, we can get the far-field scattered pressure as:

$$p = \frac{ik_0^2 c_0^2}{4} \cdot \sqrt{\frac{2}{i\pi k_0 r}} e^{ik_0 r} \left(-M + ik_0 D_x \cdot \cos\theta + ik_0 D_y \cdot \sin\theta\right) = \frac{e^{ik_0 r}}{\sqrt{r}} \cdot f\left(\theta\right) \tag{S142}$$

Here the angular dependence $f\left(\theta\right)$ of the scattered pressure fields is defined as:

$$f\left(\theta\right) = \frac{ik_0^2 c_0^2}{4} \cdot \sqrt{\frac{2}{i\pi k_0}} \left(-M + ik_0 D_x \cdot \cos\theta + ik_0 D_y \cdot \sin\theta\right) \tag{S143}$$

For any specific frequency, the scattering pattern is fully described by angular dependence $f(\theta)$; and the forward (backward) plane wave incidences are related to $\theta = 0(\pi)$. Considering the numerically derived and experimentally measured polarizability tensor, we can directly obtain the normalized numerical and experimental angular dependence of the scattered fields for Fig. 4 in the main text and Fig. S3 in the supplementary Information.

The total scattering width with the length unit in 2D space is the analogue of the total scattering cross section with the area unit in 3D space. It is defined by the integration of the differential scattering cross section, which specifies the amount of scattered sound power from a given irradiance (power per area/length). In the 2D cylindrical coordinates, we have:

$$\sigma^{sc} = \int_0^{2\pi} |f(\theta)|^2 \, d\theta \tag{S144}$$

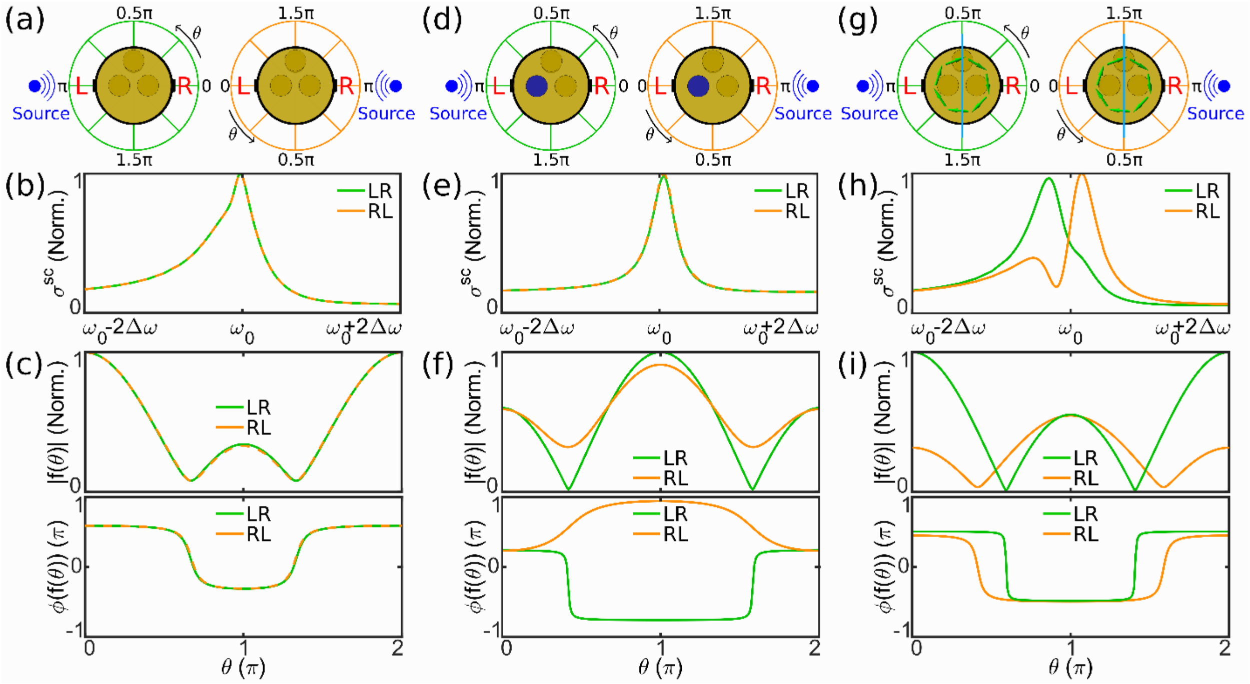

Fig. S3| Sound scattering and Willis coupling. a, Schematics of the scattering scenarios for a symmetric scatterer: an incident wave from the left (green case) and right (orange case) ports**. b,** Numerical simulation of the total scattering width for an incident wave from the left (green line) and right (orange line)**. c,** Corresponding angular dependence of the field scattered in absolute value (top) and phase (bottom). **d, e, f,** Similar to **a, b, c** but for an even Willis scatterer. **g, h, i,** Similar to **a, b, c** for a purely odd Willis scatterer. The angular dependence of the scattered field is shown at resonance for the first two cases. For the odd Willis case, it is shown at the frequency for which the contrast between (LR) and (RL) total scattering cross-sections is maximum.
